# A novel smartphone app to change risk behaviors of women after gestational diabetes: A randomized controlled trial

**DOI:** 10.1371/journal.pone.0267258

**Published:** 2022-04-27

**Authors:** Anne L. Potzel, Christina Gar, Friederike Banning, Vanessa Sacco, Andreas Fritsche, Louise Fritsche, Karsten Müssig, Laura Dauben, Jochen Seissler, Andreas Lechner

**Affiliations:** 1 Diabetes Research Group, Medizinische Klinik und Poliklinik IV, Klinikum der Universität München, Munich, Germany; 2 CCG Type 2 Diabetes, Helmholtz Zentrum München, Munich, Germany; 3 German Center for Diabetes Research (DZD), Neuherberg, Germany; 4 Institute for Diabetes Research and Metabolic Diseases of the Helmholtz Zentrum München at the Eberhard-Karls-University of Tübingen, Tübingen, Germany; 5 Institute for Clinical Diabetology, German Diabetes Center, Leibniz Institute for Diabetes Research, Heinrich Heine University, Düsseldorf, Germany; 6 Division of Endocrinology and Diabetology, Medical Faculty, Heinrich Heine University, Düsseldorf, Germany; 7 Department of Internal Medicine, Niels-Stensen-Kliniken, Franziskus-Hospital Harderberg, Georgsmarienhütte, Germany; University of Colorado School of Medicine, UNITED STATES

## Abstract

**Aims:**

Women after gestational diabetes mellitus (GDM) are a risk group for cardiometabolic diseases but are hard to reach by conventional lifestyle programs. Therefore, we tested whether a novel, smartphone-delivered intervention, *TRIANGLE*, is accepted by women after GDM and alters cardiometabolic risk behaviors and outcomes. *TRIANGLE* targets gradual habit change of mind and emotion, physical activity, nutrition, and sleep.

**Methods:**

We conducted a 6-month multicenter, randomized-controlled trial of *TRIANGLE* versus standard care with 66 women 3–18 months after GDM in Germany. The primary outcome was the proportion of women achieving ≥3 out of 5 Diabetes Prevention Program goals, i.e. physical activity ≥150 min/week (moderate to high intensity), fiber intake ≥15 g/1,000 kcal, fat intake <30% of total energy intake, saturated fat intake <10% of total energy intake, and weight reduction ≥5% if BMI ≥23 kg/m^2^ or weight maintenance if BMI <23 kg/m^2^. Intervention participants also rated the *TRIANGLE* app in the Mobile Application Rating Scale (uMARS).

**Results:**

In the predefined, modified intention-to-treat analysis including 64 women, 6 out of 27 women in the intervention group [22%(10–40)] and 3 out of 27 women in the control group [11%(3–27)] reached the primary outcome (p = 0.47). In the predefined per-protocol intervention subgroup, the proportion was 4 out of 14 women [29%(11–55); p = 0.20 vs. control]. *TRIANGLE* app users were active on 42% of days and rated the app’s quality and perceived impact with 4.3±0.8 out of 5 uMARS points.

**Conclusions:**

This first trial did not show the efficacy of the *TRIANGLE* intervention. However, the app was well accepted and considered helpful by most users. Therefore, this trial supports further development and testing of *TRIANGLE* and other app interventions for women after GDM. Additionally, it identifies necessary adaptations in trial design to better accommodate non-intensive lifestyle interventions for this target group.

**Trial registration:**

Trial registration at drks.de (DRKS00012996).

## Introduction

With an estimate of 18 million pregnancies with a live birth affected worldwide in 2017, gestational diabetes mellitus (GDM) remains the most common complication during pregnancy [1]. A history of GDM indicates a tenfold higher risk for type 2 diabetes later in life [2] and an increased risk for related cardiometabolic disturbances [3] starting in the years following delivery [4]. Therefore, GDM is an indication for future health risks and, therefore, a signal to initiate preventive interventions to reduce such risks.

Several programs have attempted health-promoting lifestyle changes in mothers post-GDM, yet they achieved a limited success due to challenges specific to this target group [5, 6]. These challenges include the role and priorities as a mother, lack of social support, high demands of life, contradicting personal preferences or negative experiences aiming for a healthy lifestyle, low risk perception, limited finances and resources, and undesirable intervention formats [6]. Thus, previous intervention effectiveness has been modest [5] and even exposure to intervention content has remained limited [7].

To improve on previous programs, we designed a smartphone-based lifestyle intervention for women with recent GDM with the specific needs of young mothers in mind [8]. We applied the method of Intervention Mapping [9] to systematically create a theory- and evidence-based intervention [9] with proven behavior change methods [8] that also address lifestyle behaviors beyond nutrition and physical activity [10]. We chose a mobile health (mHealth) solution to deliver the intervention because of the common use of smartphone apps for health purposes by women post-GDM [11]. Additionally, such apps provide a practical and accessible tool for individual, self-paced, home-based, and affordable behavior change support that is compatible with daily family life, as desired by women post-GDM [6, 12]. Further, mHealth solutions offer low delivery costs, international dissemination, multimedia options, and easy integration of behavior change methods [13, 14]. Finally, subjective and objective data on app usage and perceived app quality offer detailed feedback and possibilities to rapidly adapt and improve an mHealth intervention.

The primary goal of our app-based *TRIANGLE* intervention is to support women on a daily basis to change their lifestyle habits. Intervention Mapping fostered a multi-theory approach at the habit-goal interface [15]. This approach helped us translate multiple behavior change methods into technical app features while complying with industrial standards for interactive smartphone apps and with the specific needs of women in the late postpartum phase [15].

This randomized trial collected first data on clinical effects, usage, and acceptance of the *TRIANGLE* intervention among women 3–18 months post-GDM. We also gathered user feedback on novel intervention components including habit change, psychosocial wellbeing, and sleep. In a next step, we investigated the regular users as a predefined per-protocol group. The aim was to check if this intervention subgroup matched the intended usage for this new app in real life and/or showed the expected clinical effects. Further, we wanted to analyze how these regular users differed from the irregular users.

## Research design and methods

### Study design

The *Test TRIANGLE Study* was a multicenter, 2-arm, randomized controlled trial with women 3–18 months post-GDM. In addition to the Medical Center of the Ludwig-Maximilians-Universität in Munich (main study site), the German Diabetes Center in Düsseldorf and the Institute for Diabetes Research and Metabolic Diseases in Tübingen contributed to this study. Participants were randomized into the intervention or control group in a 1:1 ratio via Randoulette version 3.1 (Institute of Biometry and Epidemiology, Munich, Germany), stratified by study center. All study participants gave written informed consent. In addition, participants in the intervention arm signed the *TRIANGLE* app’s data privacy statement, as approved by the data protection officer of the Medical Center of the Ludwig-Maximilians-Universität. The study was approved by the ethics committee of the Medical Faculty of the Ludwig-Maximilians-Universität (reference number: 17–311) and by the respective local ethics committees of the German Diabetes Center in Düsseldorf and of the Institute for Diabetes Research and Metabolic Diseases in Tübingen. It further conforms to the European Medicines Agency Guidelines for Good Clinical Practice.

### Study participants

Participants were primarily recruited by phone from the patient base of each study site from June 2017 to May 2018. The inclusion criteria were a validated diagnosis of GDM by a medical doctor in the last pregnancy according to the German guidelines [16], delivery 3–18 months prior to study inclusion, postnatal core muscle recovery, ownership of an iPhone 5 to 7 Plus, and fluent German skills. Exclusion criteria comprised: age <18 years, current or planned pregnancy during the study period, cardiopulmonary disease or restrictions in the locomotor system contraindicating a sports intervention, gastrointestinal disease contraindicating a nutrition intervention, psychiatric disease requiring therapy, other serious illnesses contraindicating a lifestyle intervention according to the principal investigator, planned inpatient hospital stay, alcohol or drug abuse, planned lifestyle changes in the areas of nutrition, physical activity, and psychosocial wellbeing apart from the *TRIANGLE* intervention, antidiabetic drug treatment or diabetes mellitus diagnosis according to the ADA criteria. The reasons to exclude patients with a diagnosis of manifest diabetes mellitus included concerns regarding the purely lifestyle-based, non-pharmacological character of the intervention.

### Intervention

**Fig 1:**
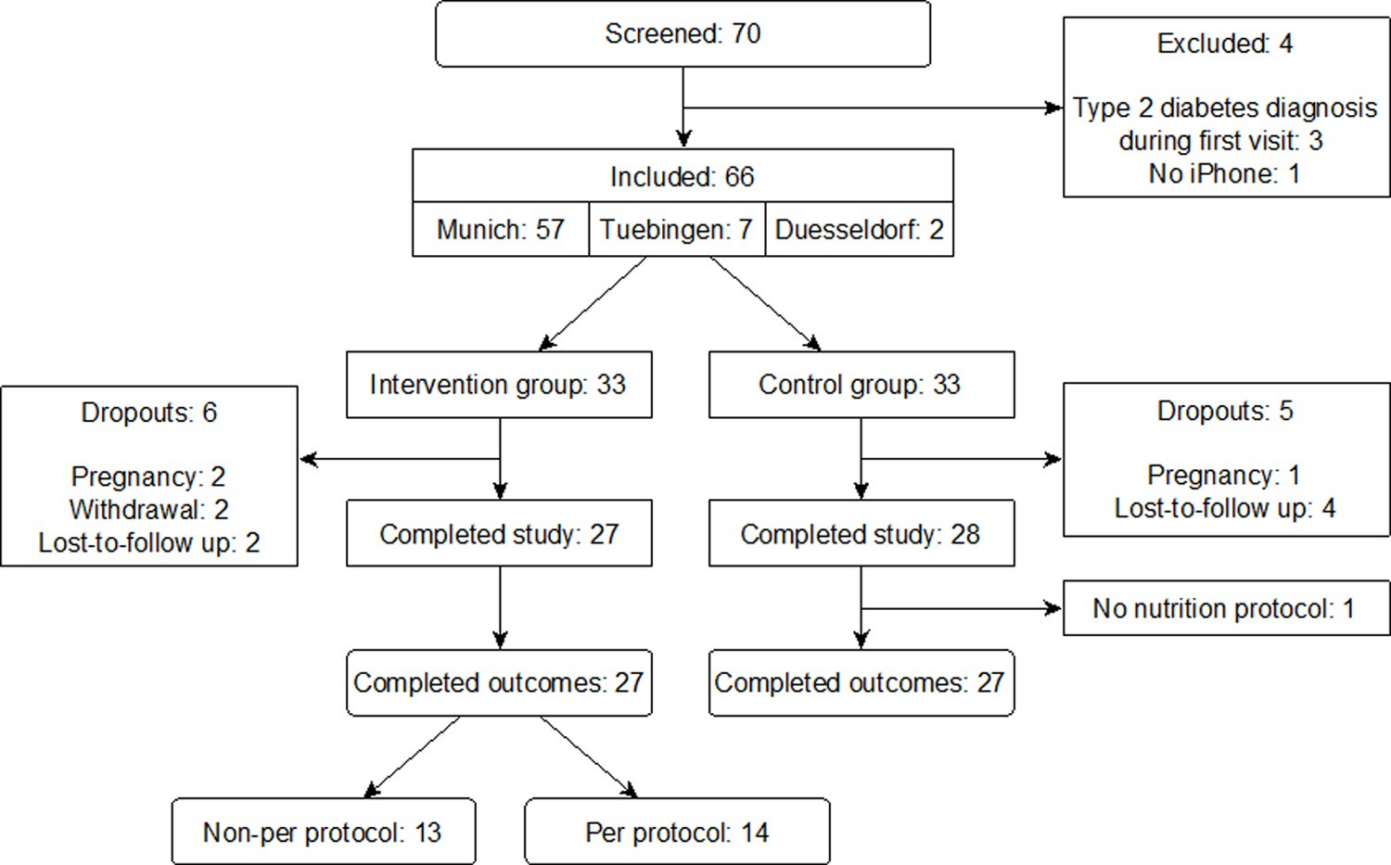
Screenshots of the *TRIANGLE* app core features. From left to right: challenge system with activity screen based on active challenges, coaching chat with in-app questionnaire, and library with exemplary article.

The self-paced 6-month *TRIANGLE* intervention for the iPhone addressed habits of mind and emotion, physical activity, nutrition, and sleep. Habit change of mind and emotion focused on establishing habits that help with decreased stress perception, realistic optimism, increased self-efficacy, and increased psychosocial wellbeing–for example by replacing automatic negative thoughts, strengthening positive emotions, training effective problem solving, and engaging into joint recreational activities. The three core features of the app (**Fig 1**)–an interactive and individualized challenge system, chat-based coaching, and a library–incorporated 39 behavior change methods. A coaching platform for healthcare practitioners mirrored these features in a content management system for individualization (**S1 Fig**). Details of the intervention can be found in a separate paper describing the planning and development process as well as early user testing of the *TRIANGLE* intervention [15]. Software support and online coaching for intervention participants were based in Munich. Control participants received a flyer with a summary of the lifestyle changes for diabetes prevention as addressed in the *TRIANGLE* intervention [15], thus adding the psychosocial wellbeing and sleep themes to usual standard care.

### Outcomes

The primary outcome was the proportion of participants reaching <3 vs. ≥3 out of 5 DPP intervention goals at visit 2 (V2), i.e. physical activity of moderate to high intensity for ≥150 min/week, dietary fiber intake ≥15 g/1,000 kcal, percent fat intake <30% of total energy intake, percent saturated fatty acid intake <10% of total energy intake, and body weight reduction ≥5% if BMI is ≥23 kg/m^2^ or body weight maintenance if BMI is <23 kg/m^2^ (**S1 Table**). For the prespecified secondary outcomes, we calculated the difference (Δ) between visit 1 (V1) and V2 for the area under the glucose curve (AUC glucose), the Insulin Sensitivity Index (ISI), the Disposition Index (DI), the peak oxygen uptake (VO_2_peak) in cardiopulmonary exercise testing, the body fat mass [kg], the World Health Organization-5 Well-Being Index (WHO-5), and the Perceived Stress Scale-10 items (PSS-10) score.

### Data collection and handling

Data collection comprised two visits (V1, V2) with questionnaires, nutrition protocols, clinical assessments, and app user logs between V1 and V2 (**S2** and **S3 Tables**). V2 were conducted between March 2018 and May 2019. Questionnaires at V1 included: medical history, family history, current diseases and medication, smoking or previous smoking, quality of life including restorative sleep measured with the WHO-5 [17], perceived stress assessed via the PSS-10 [18], and physical activity quantified by the International Physical Activity Questionnaire (IPAQ, 2002) [19]. Questionnaires at V2 comprised changes in medication since V1, illnesses since V1, adverse events, WHO- 5, IPAQ, PSS-10, and the additional question “Have you changed health-related habits in the past six months?” (“definitely yes”, “rather yes”, “rather not”, and “definitely not”). In addition, the intervention group received the user version of the Mobile Application Rating Scale (uMARS) [20].

The nutrition protocols contained four days of food and drink journaling at the time of consumption [21]. All nutrition protocols were entered into the Software PRODI® 6 Basis (Nutri-Science GmbH, Hausach, Germany) by a blinded nutritionist. The software analyzed the daily mean calorie intake (kcal), fiber intake (g), total fat intake (g), and saturated fat intake (g) per participant.

The 5-point 75-g oGTT, anthropometrics, and clinical measurements were conducted as previously described for the PPSDiab Study [22]. In case of diabetes mellitus diagnosed during V1, a participant was excluded from the study. We calculated the AUC glucose during the oGTT with the trapezoidal method [23] and the ISI according to Matsuda and De Fronzo (ISI = 10,000/√[fasting glucose x fasting insulin x (mean glucose x mean insulin)]) [24]. Further, the rise in serum insulin during the first 30 min of the oGTT (Δ ins 30’) was calculated [25] as basis for the DI (Δ ins 30’ x ISI). The stepwise ergospirometry on a bicycle ergometer (MasterScreen CPX, CareFusion, Höchberg, Germany) was conducted as described for the PPSDiab Study [26].

At V1, intervention participants answered an initial paper and pencil questionnaire for personalization purposes of the program and received a Garmin vívosmart HR® fitness tracker, a step tread, the *TRIANGLE* intervention paper note pad, and an individual login code for the *TRIANGLE* app (**S2 Table**) [15]. The client-server system tracked their activity in the *TRIANGLE* app (**Fig 1** and **S3 Table**). All *TRIANGLE* app data were encrypted and stored on the University Medical Center’s server in Munich.

### Statistics

The power calculation was based on a binary outcome: the proportion of participants reaching <3 vs. ≥3 out of 5 DPP intervention goals at V2 (**S1 Table**). Based on the MAGDA trial [7], we assumed a success rate of 15% in the control group and aimed for a 50% success rate in the intervention group–under the premises that an mHealth program will lead to both a higher exposure to intervention materials and a higher program adherence of women post-GDM. With an uncorrected chi-square test with a significance level of 5% (2-sided) and a power of 90%, 27 participants in each group were calculated. We assumed a relatively low dropout rate of 15% due to the limited 6-months duration of the trial. Hence, 32 participants needed to be randomized per group.

Categorial variables were presented as counts (n) and percentages, normally distributed metric variables as means ± standard deviations, and non-normally distributed metric variables as medians with interquartile ranges. The comparison between groups was made by using a Chi-Square test, Fisher-Exact Test or Mann-Whitney-U test. P-values <0.05 were considered statistically significant. We analyzed and visualized all data with the SAS statistical software package version 9.4 (SAS Institute Inc., Cary, USA) and Tableau Desktop 2019.3 (Tableau Software, LLC, Seattle, USA).

The primary analysis was based on all randomized participants with complete primary outcomes at V2 (predefined, modified intention-to-treat analysis). Of two predefined per-protocol groups, only per-protocol group 2 (participants who used each of the app’s core features ≥1/month throughout the study period) was analyzed since the rest of the intervention participants matched the criteria for per-protocol group 1 (participants who used the app’s core features ≥1x in total).

## Results

### Baseline characteristics

**Fig 2:**
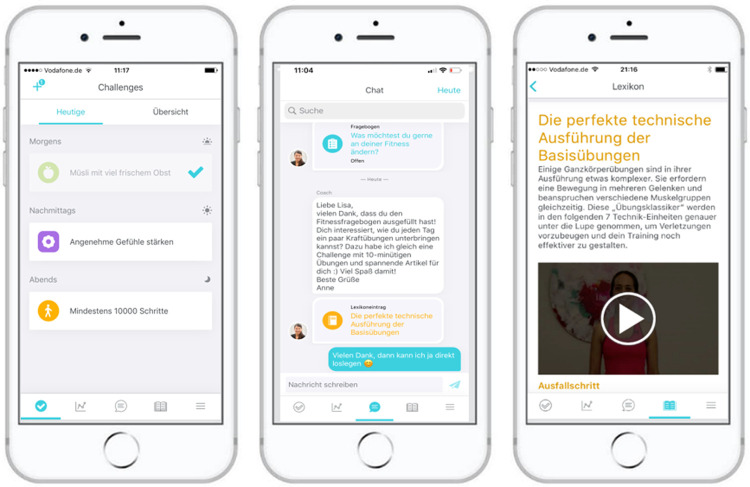
Participant flow chart for the Test *TRIANGLE* Study.

In total, 66 out of 70 screened participants were included in the study (**Fig 2**). The control and the intervention group did not differ significantly at baseline, except for more native speakers in the intervention compared to the control group (**Table 1** and **S5 Table**). The predefined, modified intention-to-treat (mITT) analyses contained 27 participants in both the intervention and the control group. In the intervention arm, the proportion of women treated with insulin during the preceding pregnancy was higher in the mITT group than in the respective dropout group. Beyond that difference, the mITT group and the dropout group were comparable in both arms (**S4 Table**).

**Table 1:** Baseline characteristics of the control and intervention group Test *TRIANGLE* Study.

		Control	Intervention	p-value
N		33	33	
**Age [years]**		35.7±4.4	37.0±3.1	0.18
**Insulin for GDM during pregnancy**		14 (42.4%)	14 (42.4%)	1.00
**Family history of diabetes**		12 (36.4%)	8 (24.2%)	0.28
**German as native language**		21 (63.6%)	29 (87.9%)	***0*.*02***
**Highest degree**	Secondary school	4 (12.1%)	6 (18.1%)	0.57
	A-levels	8 (24.2%)	5 (15.2%)	
	University	21 (63.6%)	22 (66.7%)	
**Active smoker**		2 (6.1%)	2 (6.1%)	1.00
**Currently in a job**		3 (9.1%)	8 (24.2%)	0.10
**Current breastfeeding**	No	13 (39.4%)	8 (24.2%)	0.42
	Partial	11 (33.3%)	14 (42.4%)	
	Full	9 (27.3%)	11 (33.3%)	
**Oral contraceptive use**		3 (9.1%)	3 (9.1%)	1.00
**Time since delivery [months]**		6 (5–8)	6 (5–10)	0.74
**Sectio caesarea**		15 (45.5%)	10 (30.3%)	0.20
**Physical activity [min/week]**		900 (580–1560)	920 (540–1530)	0.95
**Body weight [kg]**		72.5 (66.6–84.5)	67.6 (61.9–78.2)	0.21
**Body fat mass [kg]**		28.3 (20.2–35.0)	22.2 (17.8–30.6)	0.21
**BMI [kg/m^2^]**		27.3 (23.9–30.3)	26.0 (22.6–28.9)	0.66
**BMI category**	≥23	27 (81.8%)	22 (66.7%)	0.16
	<23	6 (18.2%)	11 (33.3%)	
AUC glucose [mg/dl*min] missing = 1		35488±8100	33126±6535	0.18
**Disposition index (DI) missing = 5**		209 (124–280)	248 (180–312)	0.13
**Insulin sensitivity index (ISI) missing = 5**		5.1 (1.9–6.7)	4.9 (3.8–6.2)	0.59
**VO_2_peak [ml/min] missing = 13**		1946±410	1924±344	0.83
**WHO-5 score**		14.2±3.4	14.2±3.9	0.70
**WHO-5 score <13**		12 (36.4%)	8 (24.2%)	0.28
**PSS-10 score missing = 1**		15.4±5.9	13.9±5.9	0.24
**Glucose tolerance status missing = 1**	Normal	19 (57.6%)	20 (62.5%)	0.42
	IFG	2 (6.1%)	5 (15.1%)	
	IGT	9 (27.2%)	6 (18.3%)	
	IFG+IGT	3 (9.1%)	1 (3.0%)	

n (percent) for categorial variables, mean±standard deviation for normally distributed metric variables, median (first and third quartile) for other metric variables; Chi-Square or Fisher-Exact Test for categorial and Mann-Whitney-U Test for metric variables.

The per-protocol group contained 14 out of the 27 women analyzed in the intervention arm. This group did not differ significantly from the non-per-protocol group (13 women, **S4 Table**) or from the control group (27 women, **S5 Table**).

### Primary outcome

The primary outcome (≥3 out of 5 DPP points) was achieved by 3 women [11%(3–27)] in the control group and by 6 women [22%(10–40)] in the intervention group (**Table 2**; p-value 0.47). Neither the binary DPP score as the primary outcome nor its individual lifestyle components differed significantly between the control and the intervention group.

**Table 2:** Group comparisons in the primary and secondary outcomes between the control, intervention, and per-protocol group in the Test *TRIANGLE* Study.

		Control	Intervention	p-value ^a)^	Per-protocol	p-value ^b)^
N		27	27		14	
**Primary outcome**						
**DPP score at V2**	0–2 pts.	24 (89%)	21 (78%)	0.47	10 (71%)	0.20
	3–5 pts.	3 (11%)	6 (22%)		4 (29%)	
**Single components of the primary outcome**						
**Physical activity at V2 [min/week]**		743 (360–1,340)	680 (520–1,125)	0.87	868 (375–1,125)	0.97
**Δ physical activity V1 to V2 [min/week]**		-108 (-433-76)	-70 (-850-190)	0.95	-58 (-578-429)	0.65
**Fiber intake at V2 [g per 1,000 kcal]**		8 (7–11)	10 (8–12)	0.14	12 (10–13)	***0*.*007***
**Δ fiber intake V1 to V2 [g per 1,000 kcal] missing = 1**		-1.6 (-3.6–2.0)	+0.9 (-1.8–3.3)	0.13	+2.7 (0.2–5.3)	***0*.*03***
**Fat intake at V2 [% of total kcal]**		39 (31–42)	36 (33–42)	0.99	36 (32–42)	0.78
**Δ fat intake V1 to V2 [% of total kcal] missing = 1**		-1.9 (-4.9–2.5)	-1.3 (-4.6–5.7)	0.44	+1.3 (-8.6–5.8)	0.57
**Saturated fat intake at V2 [% of total kcal]**		13 (11–16)	13 (10–14)	0.39	11 (10–14)	*0*.*10*
**Δ saturated fat intake V1 to V2 [% of total kcal] missing = 1**		+0.1 (-3.3–2.6)	-0.8 (-2.0–2.3)	0.57	-1.5 (-2.6–1.2)	0.31
**Δ body weight V1 to V2 [% of baseline]**	BMI ≥23	-1.4 (-4.3–2.4)	-1.4 (-5.6–2.5)	0.76	-3.9 (-9.4–0.9)	0.22
	BMI <23	-0.6 (-1.2–0.3)	+0.3 (-2.5–0.8)	0.44	0.3 (-2.8–0.9)	0.42
**Prespecified secondary outcomes**						
Δ AUC glucose V1 to V2 [mg/dl*min] missing = 3		-752 ±6,475	-2,328 ±5,779	0.76	-4,110 ±6,972	0.25
**Δ ISI V1 to V2 missing = 7**		-0.1 (-1.9–0.9)	-0.3 (-0.7–0.3)	0.93	0.0 (-0.3–0.7)	0.50
**Δ DI V1 to V2 missing = 7**		-11 (-49-93)	+3 (-54-40)	0.43	+8 (-45-39)	0.52
**Δ VO_2_peak V1 to V2 [ml/min] missing = 15**		0 (-132-119)	+15 (-137-190)	0.51	+102 (-70-269)	*0*.*10*
**Δ body fat mass V1 to V2 [kg] missing = 1**		-0.5±3.7	-0.6±2.5	0.96	-0.9±2.6	0.74
**Δ WHO-5 score V1 to V2 missing = 1**		0.0 (-3.0–3.0)	+1.0 (-2.0–3.0)	0.29	+1.5 (-2.0–4.0)	0.19
**Δ PSS-10 score V1 to V2 missing = 1**		-1.5 (-4.0–3.0)	-1.0 (-3.0–3.0)	0.53	-3.0 (-4.0–0.0)	0.21
**Additional analyses**						
**Glucose tolerance status at V2**	NGT	14 (51.9%)	15 (55.6%)	0.70	10 (71.4%)	0.69
	IFG	7 (25.9%)	8 (29.6%)		3 (21.4%)	
	IGT	3 (11.1%)	4 (14.8%)		1 (7.1%)	
	IFG+IGT	1 (3.7%)	0 (0.0%)		0 (0.0%)	
	type 2 diabetes	2 (7.4%)	0 (0.0%)		0 (0.0%)	
**“Have you changed health-related habits in the past six months?” missing = 2**	Def. not	6 (22.2%)	1 (4.0%)	***<0*.*0001***	1 (7.7%)	***0*.*002***
	Rather not	11 (40.7%)	0 (0.0%)		0 (0.0%)	
	Rather yes	8 (29.6%)	13 (52.0%)		7 (53.9%)	
	Def. yes	2 (7.4%)	11 (44.0%)		5 (38.5%)	

n (percent) for categorial variables, mean±standard deviation for normally distributed metric variables, median (first and third quartile) for other metric variables; ^a, b)^ Chi-Square or Fisher-Exact Test for categorial and Mann-Whitney-U Test for metric variables; p-value ^a)^ for comparison of control and intervention subjects (modified intention-to-treat group); p-value ^b)^ for comparison of control and per-protocol subjects. The per-protocol group is a subset of participants of the intervention group; DPP = Diabetes Prevention Program, V1 = visit 1, V2 = visit 2.

### Secondary outcomes

The prespecified secondary outcomes did not differ significantly between the control and the intervention group (**Table 2**). In further secondary analyses, two women in the control group developed type 2 diabetes during the study while no woman in the intervention group developed type 2 diabetes (p-value 0.7; **Table 2**). In the intervention group, 24 women (96%) stated that they had changed health-related habits during the study versus 10 women (37%) in the control group (p <0.0001; bottom of **Table 2**). No adverse events were causally linked to the intervention.

### Per-protocol group

The primary outcome (≥3 out of 5 DPP points) was achieved by 4 women [29%(11–55) in the per-protocol group (**Table 2**; p-value 0.20 when compared with the control group). In the per-protocol group, fiber intake at V2, as well as the change in fiber intake from V1 to V2, were significantly higher compared to the control group (**Table 2** and **S5 Table**). Further, the per-protocol group showed non-significant, yet clinically relevant changes towards less saturated fat intake, a higher VO_2_peak, a higher weight loss in the overweight subgroup, a higher WHO-5 score and a lower PSS-10 score (**Table 2**).

### Process measures of the intervention

**Fig 3:**
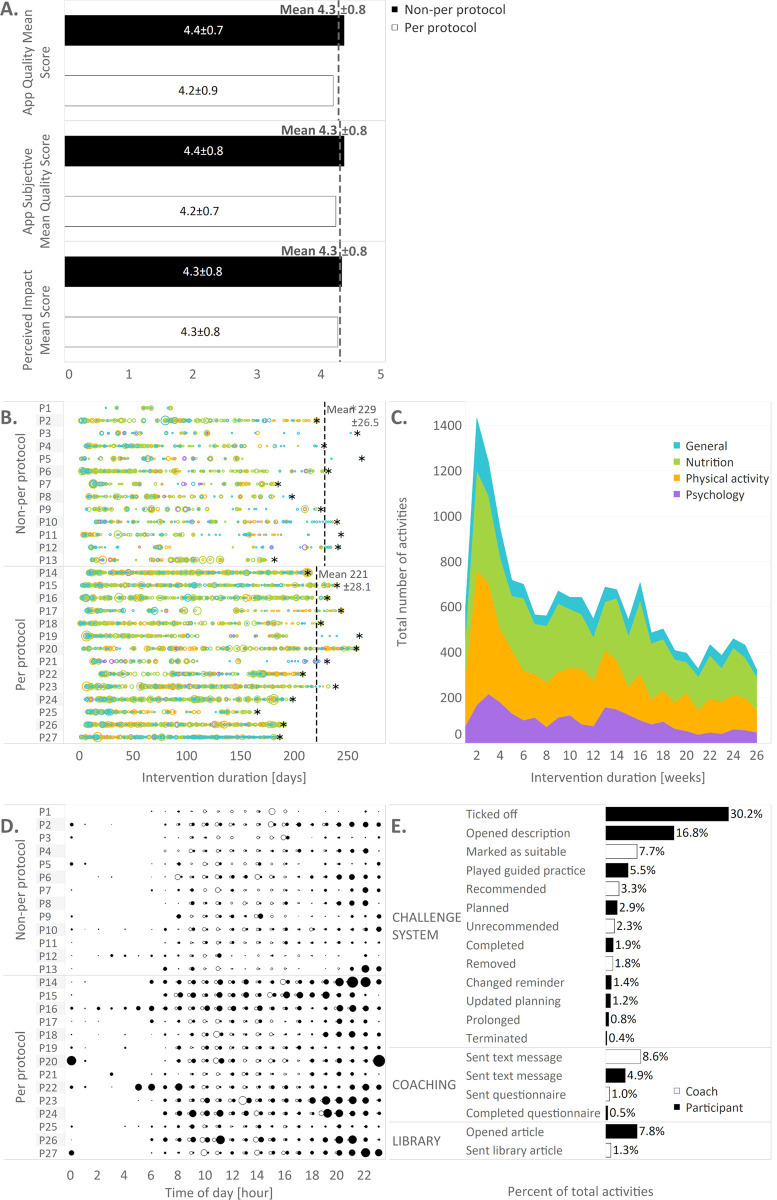
App acceptance and usage in the Test *TRIANGLE* Study. N = 27, A. Results of the user Mobile Application Rating Scale (uMARS), black bars = non-per-protocol group (n = 13); white bars = per-protocol group (n = 12, two missing); all values as mean±standard deviation, B. Number of app activities per participant over time; n = 13 in the non-per-protocol group (top) and n = 14 in the per-protocol group (bottom); one circle per active day, circle size reflects number of activities, asterisk marks visit 2, P = participant; C. Proportion of total number of app activities over time stratified by theme D. Number of app activities per participant per time of the day, n = 13 in the non-per-protocol group (top) and n = 14 in the per-protocol group (bottom); black circles = participant activity, white circles = coach activities, one circle per active hour, circle size reflects number of activities, P = participant, E. Percent of app activities per sub-feature, black bars = participant, white bars = coach.

*TRIANGLE* app users rated the app with a mean of 4.3 out of 5.0 points (n = 25) on all main uMARS scores (the App Quality Mean Score, the App Subjective Mean Quality Score, and the Perceived Impact Mean Score) (**Fig 3A**). The per-protocol group and the non-per-protocol group evaluated the *TRIANGLE* app comparably (**Fig 3A**).

Each participant used the app for each lifestyle area at some point (**Fig 3B**). About half of the participants (per-protocol group, n = 14) used the app regularly throughout the study, some on an almost daily basis. On average, the app was available for participants on 224±27 days and used on 93±48 days, corresponding to 42% and to 3.7±0.5 active days/week. In the per-protocol group, the mean of available days was 221±28.1 days, with a mean of 4.5±0.7 active days/week while in the non-per-protocol group, the mean of available days was 229±26.5, with a mean of 2.7±0.6 active days/week. The mean for app activities per active day was 7.5±1.5 in the per-protocol group and 6.3±1.9 in the non-per-protocol group. App activity over time varied per participant (**Fig 3B**).

Total app activities peaked between week 2–3, before dropping until week 8 (**Fig 3C**). Weeks 9–16 marked a plateau, before activity dropped to a lower level to reach another plateau around week 22. Nutrition was accessed the most over time, followed by physical activity. Overall, the lifestyle areas were used continuously over 6 months (**Fig 3C**).

Participants used the app at any time of the day and independently from coaching times (**Fig 3D**). Participants were most active in the app between 9 am and 11 am, and between 8 pm and 10 pm while the coach was most active between 8 am and 4 pm.

In sum, activities by the coach were low in comparison to app activities by participants (**Fig 3E**). Higher coaching activity was not associated with higher activity by participants. Regarding the sub-features of the app, participants primarily ticked off challenges, followed by opened challenge descriptions, opened library articles, guided practice, and sent text messages (**Fig 3E**). The coach primarily sent text messages, including motivational messages, and marked challenges as suitable or recommended.

## Discussion

This first, randomized trial of the novel *TRIANGLE* intervention for women post-GDM did not demonstrate a significant intervention effect in the intention-to-treat analysis. The main reasons for this result were likely an insufficient statistical power and the 6-month intervention period, which was too short for the chosen non-intensive intervention in combination with the chosen outcomes. The app intervention was well accepted and deemed effective by its users. This finding and clinically relevant changes in the intervention group suggested that a larger and longer trial, after some adaptations of the *TRIANGLE* intervention, could demonstrate statistically significant, clinical effects in the future.

### No significant outcome but clinically relevant and subjective effects of intervention

The proportion of women reaching the primary outcome was double in the intervention (22%) compared to the control (11%) group–albeit not significantly different. We had chosen an ambitious binary DPP score as the primary outcome after six months due to the assumption of an enhanced program exposure because of the smartphone app delivery. While enhanced exposure over traditional intervention approaches was achieved, persistent program adherence and clinical effects were only demonstrated in a subgroup of intervention participants. Nevertheless, 96% of women in the intervention group indicated that they changed health-related habits during the trial versus 37% in the control group. Hence, targeted habits changed during the intervention but either the sum of small habit changes or the duration until they result into clinically relevant effects were insufficient. This points to necessary adaptions of the intervention as well as the study design, which should focus on the aim of long-term health habit changes that match the non-intensive approach.

Other intervention trials with women post-GDM also did not find significant intervention effects for the DPP outcomes [7, 27]. This further supports the notion that other and/or broader surrogate outcomes should be examined, as suggested by similar studies [28–30]. Meta-analyses with pooled data on intervention trials post-GDM that did not consider the DPP cutoffs showed significant differences between the intervention and control groups for body weight loss [5, 8, 31]. Thereby, studies with a duration of more than one year reached a significantly higher weight loss compared to those with a duration below one year [5]. Hence, a combination of objective clinical parameters such as changes in body weight, body fat mass, ISI, and peak oxygen uptake [32] and other surrogate outcomes such as experience sampling [33] to detect changes in habits should be further explored in this context. The current work of an international consortium defining a core outcome set for intervention studies post-GDM will give more insights into the best suited outcome measures for this scenario [34].

### Stronger intervention effects in the predefined per-protocol group

We predefined and analyzed the subgroup of regular app users in a per-protocol group to characterize these users and to compare the results they achieved with those who did not interact with the app on a regular basis throughout the study period. The 100% proportion of native speakers in the per-protocol group highlighted the importance of language and culture for this type of intervention. Nevertheless, several intervention outcomes, in particular a significantly higher fiber intake at V2, as well as the change in fiber intake from V1 to V2, and clinically relevant changes for saturated fat intake, VO_2_peak, weight loss in the overweight subgroup, WHO-5 score and PSS-10 score in the per-protocol group compared to the control group, pointed to possible benefits of regular app use.

Higher fiber intake was a central objective of the diet change suggested in the app. Therefore, the significantly increased fiber intake in regular users is reassuring. Similarly, switching to healthier fats was promoted for all study participants–seemingly with some success in the per-protocol group. In contrast, the reduction of overall fat intake was not the main intervention target for most participants due to the higher priority of enhancing carbohydrate and fat quality, which corresponds to the absence of a consistent change in this dietary component. Similarly, weight loss was only suggested for women with a BMI of at least 23 and moderate weight reduction was achieved in the per-protocol group for this stratum of women. Finally, we could not detect a difference in physical activity between the groups at the end of the intervention, despite regular use of the exercise features of the app as well as a clinically relevant increase of peak oxygen uptake in the per-protocol group. We attribute this contradiction to the fact that the physical activity questionnaire proved to be an unsuitable measurement tool for this study population.

In the secondary outcomes, wellbeing (WHO-5) increased in a clinically relevant way in the per-protocol group and, in parallel, perceived stress (PSS-10) decreased. This points to likely changes in mental and emotional habits for those interacting with the app on a regular basis. In sum, this study suggests that the app successfully delivered the intended intervention to women in the per-protocol group, i.e. the regular users, but that intervention content and measurement tools require optimization.

### TRIANGLE app well accepted and highly rated by all users

The *TRIANGLE* app quality ratings in the uMARS with 4.3 out of 5.0 points lie within the top range of health apps rated by users [35]. When comparing the uMARS ratings of the per-protocol group with the non-per-protocol group, a more regular app activity did not affect the App Quality Mean Score, the App Subjective Quality Mean Score or the Perceived Impact Mean Score. This indicates that not only participants who used the *TRIANGLE* app on a regular basis, but also irregular users accepted the app and considered it of high quality.

### All app core features and lifestyle areas used by participants

The *TRIANGLE* app proved suitable to reach women post-GDM. On average, the app was used on 42% of the days available, corresponding to a mean of 3.7 days/week, and at any time of the day. Approximately half of the *TRIANGLE* intervention participants (n = 14, per-protocol group), used the app on 4.5 days/week–almost as intended (≥5 out of 7 days/week). Yet, further adherence-boosting strategies are needed to achieve the intended app usage of ≥5 out of 7 days/week for most users.

*TRIANGLE* app users were most active in the first month, which mirrors the usage pattern of other health apps [36]. The regular use of features in all three lifestyle areas supports a recent model for effective interventions during and following GDM that includes mental and psychosocial wellbeing [10] versus the traditional nutrition and/or physical activity approach [5, 8].

The high usage of the interactive features stressed the importance of self-monitoring, two-way communication, and counselling in health apps [37]. The low coach-to-participant activity ratio illustrated a realistic scope for implementation in routine care, as achieved for similar concepts [37]. Moreover, some of the current coaching activities, such as the motivational messages, may be automated in the future to further lower staff requirements. This study also indicated that a fully automated *TRIANGLE* app version may be suitable for some women post-GDM. Other possible features for apps post-GDM are currently being tested by other work groups, such as self-monitoring for diabetes screening, external links e.g. to a Facebook community page or automated features including a virtual health coach that guides participants through different modules [11].

### Strengths and weaknesses of the trial

The *Test TRIANGLE Study* was the first clinical trial of a smartphone app-based intervention in the post-GDM context. It permitted an initial assessment of likely clinical effects and feasibility of the *TRIANGLE* intervention via both objective (clinical parameters, user logs) and subjective (questionnaires, nutrition protocols) data. User logs in the intervention arm gave insights into the use of program components. This study can therefore guide the future development of smartphone-based interventions post-GDM and of respective trials.

The weaknesses of this study include that it was underpowered due to the underlying assumptions in the power calculation. However, this is not unusual for a first clinical trial with a novel intervention and results of this trial will guide the planning of confirmatory studies. Although equal to the median trial duration post-GDM [34], the 6-month intervention period, in retrospect, was too short for this type of intervention. Additionally, the DPP goals, chosen in analogy to previous prevention trials after GDM [38, 39], proved suboptimal for the non-intensive *TRIANGLE* intervention. Another potential weakness of this study was its rather homogenous cohort, with a possible socioeconomic bias due to the inclusion of iPhone users only, a high education level, and mostly native speakers. Yet, the characteristics of this sample are generally comparable to post-GDM cohorts such as the cohort of the PPSDiab study [25]. Future trials should attempt to recruit a more diverse group of women and the app should be provided for the Android system in the next iteration. In addition, the dropouts without V2 results may have introduced some bias–despite the similar and known reasons for dropout (see Fig 2) and a respective group-based comparison of baseline characteristics that showed no significant differences, except for fewer dropouts of women who used insulin during their preceding pregnancy in the intervention group (see S4 Table). Finally, the main study outcomes relied on subjective measurement tools (nutrition protocols, IPAQ, WHO-5, and PSS-10) that are prone to bias and better suited for large cohorts [19, 40]. This was in particular visible for the long IPAQ that led to unrealistic values for moderate to intensive physical activity both at baseline and follow-up and thus proved unsuitable in this context. Alternative, objective outcomes should, therefore, be employed in future trials and a core outcome set for behavioral interventions post-GDM should be developed, as already suggested by others [34].

### Outlook

The present study illustrates some of the opportunities and challenges of mHealth interventions for women post-GDM. It demonstrates that the *TRIANGLE* approach resonates with the participating women. Thus, mHealth is a promising way of program delivery in this context and it is suitable for large-scale implementation. However, long-term engagement remains challenging in women post-GDM who prioritize family needs over self-care. Thus, further adaptations to *TRIANGLE* seem warranted. Additionally, trial designs for such gradual interventions require further development. Longer intervention periods, alternative, objective surrogate outcomes and, eventually, assessment of hard disease outcomes are necessary.

## Supporting information

S1 Checklist CONSORT 2010 checklist of information to include when reporting a randomised trial*. (DOC)

S1 Fig TRIANGLE app and coaching platform workflow. (TIF)

S1 Table Statistical analysis plan for the primary outcome of the Test TRIANGLE Study; definition of the DPP intervention goals used in the primary endpoint. *) Current guidelines contain a threshold of a BMI ≥25 kg/m^2^ for diabetes prevention. However, we chose a threshold of BMI ≥23 kg/m^2^ since our priority population is considerably younger compared to traditional type 2 diabetes prevention cohorts. V1 = visit 1, V2 = visit 2.
(PDF)

S2 Table Overview study workflow in the Test TRIANGLE Study. GDM = gestational diabetes mellitus, oGTT = oral glucose tolerance test, V1 = visit 1, V2 = visit 2.
(PDF)

S3 Table Specification of the collected TRIANGLE app data per user. (PDF)

S4 Table Baseline characteristics with additional values for the dropouts in both the control and the intervention group, and for the per-protocol group. n (percent) for categorial variables, mean±standard deviation for normally distributed metric variables, median (first and third quartile) for other metric variables; ^a, b)^ Chi-Square or Fisher-Exact Test for categorial and Mann-Whitney-U Test for metric variables; control ITT vs. control dropouts, intervention ITT vs. intervention dropouts, per-protocol vs. non-per-protocol; all p-values ≥0.05 except for *). The per-protocol group is a subset of participants of the intervention group who used the core features of the app regularly throughout the study. AUC glucose = area under the glucose curve, DPP = Diabetes Prevention Program, IFG = impaired fasting glucose, IGT = impaired glucose tolerance, ITT = intention to treat, oGTT = oral glucose tolerance test, PSS-10 = Perceived Stress Scale-10 item, VO_2_peak = peak oxygen uptake, WHO- 5 = World Health Organization-5 Well-Being Index.
(PDF)

S5 Table Baseline values for the primary outcome and its individual components, as well as changes of these variables from visit 1 to visit 2 in the control, intervention and per-protocol group. n (percent) for categorial variables, mean±standard deviation for normally distributed metric variables, median (first and third quartile) for other metric variables; Chi-Square or Fisher-Exact Test for categorial and Mann-Whitney-U Test for metric variables; p-value ^a)^ for comparison of control and intervention subjects (modified intention-to-treat group); p-value ^b)^ for comparison of control and per-protocol subjects. The per-protocol group is a subset of participants of the intervention group who used the core features of the app regularly throughout the study. DPP = Diabetes Prevention Program, V1 = visit 1, V2 = visit 2.
(DOCX)

S1 File Study protocol (original German version). (DOCX)

S2 File Study protocol (English translation). (DOCX)
