# Supplementary figures and images for: A novel smartphone app to change risk behaviors of women after gestational diabetes: A randomized controlled trial
Source: PLoS One. 2022 Apr 27;17(4):e0267258. doi: 10.1371/journal.pone.0267258 (PMC9045614; doi:10.1371/journal.pone.0267258)

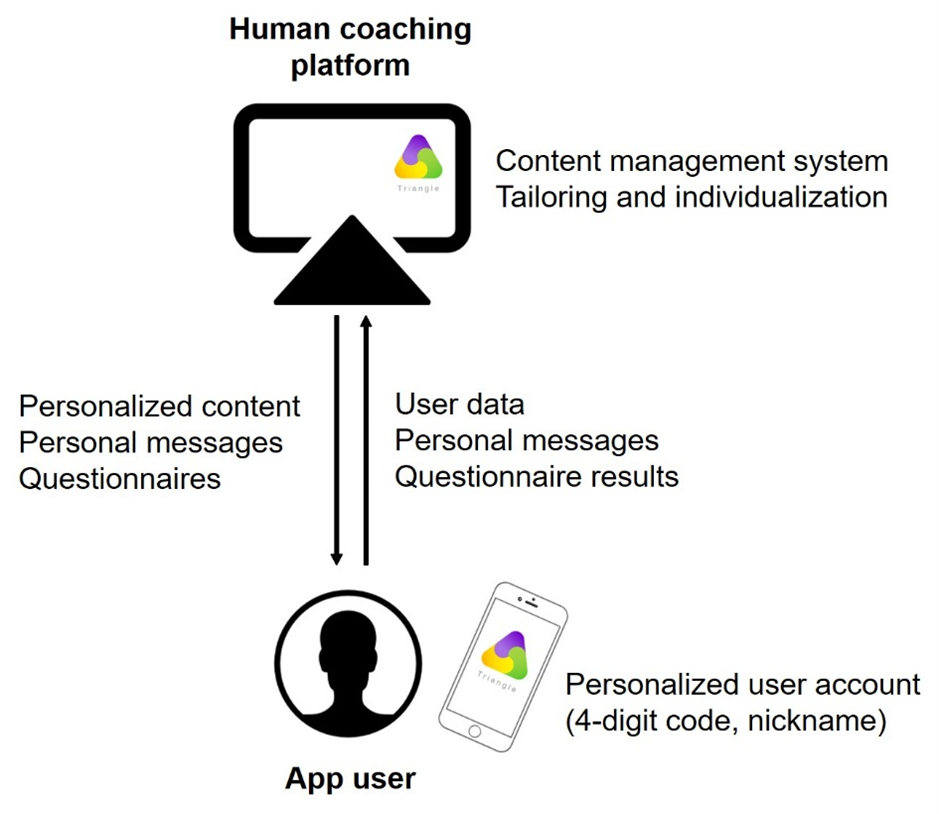

Supplement: S1 Fig — (TIF) [file pone.0267258.s002.tif]
