# Supplementary material for: A novel smartphone app to change risk behaviors of women after gestational diabetes: A randomized controlled trial
Source: PLoS One. 2022 Apr 27;17(4):e0267258. doi: 10.1371/journal.pone.0267258 (PMC9045614; doi:10.1371/journal.pone.0267258)
Supplement: S1 Table — *) Current guidelines contain a threshold of a BMI ≥25 kg/m2 for diabetes prevention. However, we chose a threshold of BMI ≥23 kg/m2 since our priority population is considerably younger compared to traditional type 2 diabetes prevention cohorts. V1 = visit 1, V2 = visit 2. (PDF) [file pone.0267258.s003.pdf]

S2 Table: Statistical analysis plan for the primary outcome of the Test *TRIANGLE* Study; Definition of the DPP Intervention goals used in the primary endpoint

Intervention goal	1 point if	Additional condition
**Increased physical activity**	≥ 150 min of moderate to high intensity per week at V2	
**Increased dietary fiber intake**	≥ 15 g per 1,000 kcal at V2	
**Decreased dietary fat intake**	≤ 30% of total energy intake at V2	
**Decreased dietary saturated fat intake**	≤ 10% of total energy intake at V2	
**Body weight management**	Body weight at V2 ≤ 95% of V1	If BMI at V1 ≥ 23 kg/m^2 *)
	Body weight at V2 ≤ 100% of V1	If BMI at V1 = 20 - 22.9 kg/m^2

*) Current guidelines contain a threshold of a BMI ≥ 25 kg/m^2 for diabetes prevention. However, we chose a threshold of BMI ≥ 23 kg/m^2 since our priority population is considerably younger when compared to traditional type 2 diabetes prevention cohorts.
V1 = visit 1, V2 = visit 2
