# Supplementary material for: A novel smartphone app to change risk behaviors of women after gestational diabetes: A randomized controlled trial
Source: PLoS One. 2022 Apr 27;17(4):e0267258. doi: 10.1371/journal.pone.0267258 (PMC9045614; doi:10.1371/journal.pone.0267258)
Supplement: S2 Table — GDM = gestational diabetes mellitus, oGTT = oral glucose tolerance test, V1 = visit 1, V2 = visit 2. (PDF) [file pone.0267258.s004.pdf]

S3 Table: Overview study workflow in the Test *TRIANGLE* Study

	**V1**	***TRIANGLE* intervention vs. standard care**	**V2**
	3-18 months post-GDM	6-8 months	6-8 months after V1
**Verification of in- and exclusion criteria**	X (prior to V1)		
**Informed consent**	X		
**Nutrition protocols**	X (at least 4 days prior to V1)		X (at least 4 days prior to V2)
**Questionnaires**	X		X
**Fasting blood sampling**	X		X
**Five-point oGTT**	X		X
**Physical examination and bioelectrical impedance analysis**	X		X
**Ergospirometry (optional)**	X		X
**Standard care lifestyle leaflet**	X (control group only)		
**Initial paper and pencil questionnaire *TRIANGLE* app, all *TRIANGLE* intervention materials**	X (intervention group only)		X (control group only)
**User logs *TRIANGLE* app**		X (intervention group only)	

GDM = gestational diabetes mellitus, oGTT = oral glucose tolerance test, V1 = visit 1, V2 = visit 2
