# Supplementary material for: A novel smartphone app to change risk behaviors of women after gestational diabetes: A randomized controlled trial
Source: PLoS One. 2022 Apr 27;17(4):e0267258. doi: 10.1371/journal.pone.0267258 (PMC9045614; doi:10.1371/journal.pone.0267258)
Supplement: S3 Table — (PDF) [file pone.0267258.s005.pdf]

S4 Table: Specification of the collected *TRIANGLE* app data per user

Technical core feature	Actor	Motion data of technical sub-feature with time stamp
**Challenge system (including progress visualization and reminders)**	Coach	• (Un-) marked challenge as suitable for user<br>• (Un-) recommended challenge for user
	User	• Opened challenge description<br>• Accepted challenge<br>• Played video or audio file in challenge<br>• Ticked off challenge (retrospectively)<br>• Undo ticked off challenge (retrospectively)<br>• Terminated challenge<br>• Prolonged challenge<br>• Completed challenge<br>• Changed reminders
**Coaching**	Coach	• Sent text message to user<br>• Sent questionnaire to user
	User	• Sent text message to coach<br>• Completed questionnaire
**Library**	Coach	• Sent library article to user
	User	• Opened library article<br>• Played video or audio file in library article
**Other**	User	• Registered with individual code
