# Supplementary material for: A novel smartphone app to change risk behaviors of women after gestational diabetes: A randomized controlled trial
Source: PLoS One. 2022 Apr 27;17(4):e0267258. doi: 10.1371/journal.pone.0267258 (PMC9045614; doi:10.1371/journal.pone.0267258)
Supplement: S4 Table — n (percent) for categorial variables, mean±standard deviation for normally distributed metric variables, median (first and third quartile) for other metric variables; a, b) Chi-Square or Fisher-Exact Test for categorial and Mann-Whitney-U Test for metric variables; control ITT vs. control dropouts, intervention ITT vs. intervention dropouts, per-protocol vs. non-per-protocol; all p-values ≥0.05 except for *). The per-protocol group is a subset of participants of the intervention group who used the core features of the app regularly throughout the study. AUC glucose = area under the glucose curve, DPP = Diabetes Prevention Program, IFG = impaired fasting glucose, IGT = impaired glucose tolerance, ITT = intention to treat, oGTT = oral glucose tolerance test, PSS-10 = Perceived Stress Scale-10 item, VO2peak = peak oxygen uptake, WHO- 5 = World Health Organization-5 Well-Being Index. (PDF) [file pone.0267258.s006.pdf]

S5 Table: Baseline characteristics with additional values for the dropouts in both the control and the intervention group and with the per-protocol group

		Control ITT	Control dropouts	Intervention ITT	Intervention dropouts	Per-protocol	Non-per-protocol
**n**		27	6	27	6	14	13
**Age [years]**		35.8±4.4	35.5±4.6	37.3±2.9	35.5±3.9	37.6±3.5	37.0±2.2
**Insulin during pregnancy**		12 (44.4%)	2 (33.3%)	**14 (51.9%)***	**0 (0%)***	10 (71.4%)	4 (30.1%)
**Family history of diabetes**		9 (33.3%)	3 (50.0%)	7 (25.9%)	1 (16.7%)	5 (35.7%)	2 (15.4%)
**German as native language**		18 (66.7%)	3 (50.0%)	24 (88.9%)	5 (83.3%)	14 (100.0%)	10 (76.9%)
**Highest degree**	Secondary school	5 (18.5%)	0 (0%)	4 (14.8%)	1 (16.7%)	3 (21.4%)	2 (15.4%)
	A-levels	5 (18.5%)	2 (33.3%)	6 (22.2%)	0 (0%)	4 (28.6%)	1 (7.7%)
	University	17 (63.0%)	4 (66.7%)	17 (63.0%)	5 (83.3%)	7 (50.0%)	10 (76.9%)
**Active smoker**		1 (3.7%)	1 (16.7%)	1 (3.7%)	1 (16.7%)	1 (7.1%)	0 (0%)
**Currently in a job**		3 (11.1%)	0 (0%)	6 (22.2%)	2 (33.3%)	5 (35.7%)	1 (7.7%)
**Current breast feeding**	No	9 (33.3%)	4 (66.7%)	6 (22.2%)	2 (33.3%)	4 (28.6%)	2 (15.4%)
	Partial	9 (33.3%)	2 (33.3%)	12 (44.4%)	2 (33.3%)	6 (42.8%)	6 (46.2%)
	Full	9 (33.3%)	0 (0%)	9 (33.3%)	2 (33.3%)	4 (28.6%)	5 (38.5%)
**Oral contra-ceptive use**		3 (11.1%)	0 (0%)	3 (11.1%)	0 (0%)	0 (0.0%)	3 (23.1%)
**Time since delivery [months]**		6 (5-8)	9 (5-14)	6 (5-11)	7 (4-10)	6 (5-16)	6 (4-8)
**Sectio caesarea**		13 (48.2%)	2 (33.3.0%)	10 (37.0%)	0 (0%)	4 (28.6%)	6 (46.2%)
**Physical activity [min/week]**		803 (580-1,620)	1,055 (435-1,310)	855 (530-1,530)	1,000 (870-1,550)	670 (435-1,455)	960 (650-1,530)
**Body weight [kg]**		72.5 (66.3-84.4)	78.4 (66.6-86.9)	72.6 (61.9-83.2)	64.7 (60.2-73.5)	66.9 (61.9 -76.5)	75.4 (62.2 -86.0)

S5 Table (continued)

		Control ITT	Control dropouts	Intervention ITT	Intervention dropouts	Per-protocol	Non-per-protocol
**n**		27	6	27	6	14	13
**Body fat mass [kg]**		28 (20-36)	30 (22-35)	22 (18-31)	22 (18-28)	21 (17-27)	28 (19-35)
**BMI [kg/m^2]**		26.3 (23.2-30.3)	27.0 (24.1-33.8)	25.4 (22.1-30.5)	23.1 (22.9-26.4)	23.8 (21.3-27.2)	28.4 (23.4-31.1)
**BMI category**	≥ 23	21 (77.8%)	6 (100%)	19 (70.4%)	3 (50%)	8 (57.1%)	11 (84.6%)
	< 23	6 (22.2%)	0 (0%)	8 (29.6%)	3 (50%)	6 (42.9%)	2 (15.2%)
**AUC glucose [mg/dl*min]**		36,196 ±7,704	32,305 ±9,822	33,185 ±6,794	32,870 ±5,819	33,092 ±7,406	33,292 ±6,330
**Disposition index (DI)**		209 (122-282)	210 (169-260)	248 (180-312)	254 (189-278)	253 (180-312)	247 (168-325)
**Insulin sensitivity index (ISI)**		4.3 (1.9-6.6)	5.8 (3.8-6.7)	4.9 (3.1-6.2)	4.9 (4.3-5.7)	4.7 (3.8-5.5)	5.6 (2.5-6.7)
**VO_2peak [ml/min]**		1,926±439	2,027±293	1,889±351	2,062±305	1,966±369	1,818±334
**WHO-5 score**		14.4±3.4	13.2±3.3	14.4±3.1	13.2±6.6	13.8±3.2	15.2±3.1
**WHO-5 score < 13**		10 (37.0%)	2 (33.3%)	6 (22.2%)	2 (33.3%)	4 (28.6%)	2 (15.4%)
**PSS-10 score**		15.3±5.8	15.7±6.9	14.2±5.3	12.7±8.5	15.5±5.1	12.8±5.2
**Glucose tolerance status**	Normal	14 (51.8%)	5 (83.3%)	16 (59.2%)	5 (83.3%)	9 (64.3%)	6 (50%)
	IFG	2 (7.4%)	0 (0%)	5 (18.5%)	0 (0%)	1 (7.1%)	4 (33.3%)
	IGT	8 (29.6%)	1 (16.7%)	5 (18.5%)	1 (16.7%)	3 (21.4%)	2 (16.7%)
	IFG+IGT	3 (11.1%)	0 (0%)	1 (3.7%)	0 (0%)	1 (7.1%)	0 (0%)

n (percent) for categorial variables, mean ± standard deviation for normally distributed metric variables, median (first and third quartile) for other metric variables; [a, b)] Chi-Square or Fisher-Exact Test for categorial and Mann-Whitney-U Test for metric variables; control ITT vs. control dropouts, intervention ITT vs. intervention dropouts, per-protocol vs. non-per-protocol; **all p-values ≥ 0.05 except for [*)]**. The per-protocol group is a subset of participants of the intervention group who used the core features of the app regularly throughout the study. AUC glucose = area under the glucose curve, DPP = Diabetes Prevention Program, IFG = impaired fasting glucose, IGT = impaired glucose tolerance, ITT = intention to treat, oGTT = oral glucose tolerance test, PSS-10 = Perceived Stress Scale-10 item, VO_2peak = peak oxygen uptake, WHO-5 = World Health Organization-5 Well-Being Index
