# Supplementary material for: A novel smartphone app to change risk behaviors of women after gestational diabetes: A randomized controlled trial
Source: PLoS One. 2022 Apr 27;17(4):e0267258. doi: 10.1371/journal.pone.0267258 (PMC9045614; doi:10.1371/journal.pone.0267258)
Supplement: S5 Table — n (percent) for categorial variables, mean±standard deviation for normally distributed metric variables, median (first and third quartile) for other metric variables; Chi-Square or Fisher-Exact Test for categorial and Mann-Whitney-U Test for metric variables; p-value a) for comparison of control and intervention subjects (modified intention-to-treat group); p-value b) for comparison of control and per-protocol subjects. The per-protocol group is a subset of participants of the intervention group who used the core features of the app regularly throughout the study. DPP = Diabetes Prevention Program, V1 = visit 1, V2 = visit 2. (DOCX) [file pone.0267258.s007.docx]

S6 Table: Baseline values for the primary outcome and its individual components, as well as change of the DDP score between V1 and V2, in the control, intervention and per-protocol group

|  |  | Control | Intervention | p-value ^a)^ | Per-protocol | p-value ^b)^ |
| --- | --- | --- | --- | --- | --- | --- |
| n |  | 27 | 27 |  | 14 |  |
| Primary outcome | | | | | | |
| DPP score at V1*  missing=1 | 0-2 pts. | 24 (92%) | 24 (89%) | 0.67 | 12 (86%) | 0.51 |
|  | 3-4 pts. | 2 (8%) | 3 (11%) |  | 2 (14%) |  |
| ∆ DDP score V1 to V2 missing = 1 | -2 pts. | 1 | 1 | 0.86 | 0 (0%) | 0.65 |
|  | -1 pt. | 1 | 2 |  | 2 (14%) |  |
|  | 0 pts. | 15 | 15 |  | 7 (50%) |  |
|  | +1 pt. | 7 | 5 |  | 3 (22%) |  |
|  | +2 pts. | 2 | 4 |  | 2 (14%) |  |
| Single components of the primary outcome | | | | | | |
| Physical activity at V1 [min/week] |  | 866  (616-1,643) | 855  (530-1,530) | 0.79 | 670  (400-1,593) | 0.38 |
| Fiber intake at V1  [g per 1,000 kcal]  missing = 1 |  | 8 (7-12) | 8 (7-11) | 0.63 | 8 (7-11) | *0.79* |
| Fat intake at V1  [% of total kcal]  missing = 1 |  | 39 (34-45) | 38 (32-43) | 0.49 | 38 (28-43) | 0.64 |
| Saturated fat intake at V1  [% of total kcal] |  | 13 (11-17) | 12 (11-15) | 0.55 | 12 (10-16) | 0.47 |
| * The 5^th^ point of the DPP score is derived from the change in body weight during the intervention. Hence, the baseline score has a maximum of 4 points. | | | | | | |

n (percent) for categorial variables, mean ± standard deviation for normally distributed metric variables, median (first and third quartile) for other metric variables; Chi-Square or Fisher-Exact Test for categorial and Mann-Whitney-U Test for metric variables; p-value a) for comparison of control and intervention subjects (modified intention-to-treat group); p-value b) for comparison of control and per-protocol subjects. The per-protocol group is a subset of participants of the intervention group who used the core features of the app regularly throughout the study. DPP = Diabetes Prevention Program, V1 = visit 1, V2 = visit 2.
