# Supplementary material for: A novel smartphone app to change risk behaviors of women after gestational diabetes: A randomized controlled trial
Source: PLoS One. 2022 Apr 27;17(4):e0267258. doi: 10.1371/journal.pone.0267258 (PMC9045614; doi:10.1371/journal.pone.0267258)
Supplement: S1 File — (DOCX) [file pone.0267258.s008.docx]

Studienprotokoll „Test Triangle“

Multizentrische, randomisierte Pilotstudie

zur Testung einer app-basierten Lebensstilintervention

für Frauen nach Schwangerschaftsdiabetes

v12.12.2017

# Hauptstudienzentrum

Studienzentrum Diabetes – Diabetes Research Group

Medizinische Klinik und Poliklinik IV

Klinikum der Universität München

Ziemssenstr. 1

80336 München

Tel: +49 (0)89 – 4400-52234

Email: studienzentrum-diabetes@med.uni-muenchen.de

# Studienleiter

PD Dr. med. Andreas Lechner

Tel: +49 (0)89 – 4400-52185

Email: andreas.lechner@med.uni-muenchen.de

# Studienkoordination

Anne Potzel (wissenschaftliche Mitarbeiterin)

Tel:  +49 (0)89 – 4400-53193

Email: [anne.potzel@med.uni-muenchen.de](mailto:anne.potzel@med.uni-muenchen.de)

Dr. med. Friederike Banning (Studienärztin)

Tel: +49 (0)89 – 4400-52159

Email: [friederike.banning@med.uni-muenchen.de](mailto:friederike.banning@med.uni-muenchen.de)

Louise Füeßl (Studienärztin)

Tel: +49 (0) 89 – 4400 52288

Email: louise.fueessl[@med.uni-muenchen.de](mailto:friederike.banning@med.uni-muenchen.de)

Vanessa Sacco (MFA)

Tel: +49 (0)89 – 4400-52234

Email: vanessa.sacco[@med.uni-muenchen.de](mailto:friederike.banning@med.uni-muenchen.de)

Janina Neubarth (wissenschaftliche Mitarbeiterin)

Tel:  +49 (0)89 – 4400-53193

Email: janina.neubarth@med.uni-muenchen.de

# Datenmanagement und Biometrie

Dipl.-Stat. Marietta Rottenkolber

Tel: +49 (0)89 – 4400-53193

Email: marietta.rottenkolber@med.uni-muenchen.de

# Weitere Studienzentren

Institut für Diabetesforschung und Metabolische Erkrankungen, Universitätsklinikum Tübingen, Klinik für Innere Medizin IV, Otfried-Müller-Straße 10, 72076 Tübingen

Universitätsklinikum Dresden, Medizinischen Klinik und Poliklinik III, Fetscherstr. 74, 01307 Dresden

Deutsches Diabeteszentrum (DDZ), Institut für Klinische Diabetologie, Leibniz Zentrum für Diabetesforschung, Heinrich-Heine-Universität Düsseldorf, Auf´m Hennekamp 65, 40225 Düsseldorf

# Klinischer Hintergrund

Der Gestationsdiabetes ist eine Störung des Zuckerstoffwechsels, die zeitlich begrenzt während einer Schwangerschaft auftritt. Bei entsprechender Behandlung ist die Prognose für Mutter und Kind bei Gestationsdiabetes sehr günstig.

Frauen, die einen Gestationsdiabetes entwickeln, haben jedoch ein deutlich erhöhtes Risiko für einen späteren Typ-2-Diabetes. Typ-2-Diabetes ist eine chronische und teils schwierig zu behandelnde Stoffwechselerkrankung mit lebensbedrohlichen Komplikationen. Die Prävalenz von Typ-2-Diabetes 10 Jahre nach einem Gestationsdiabetes liegt bei 20 bis 70% (1; 2).

Lebensstilinterventionen, wie Gewichtsreduktion, eine Ernährungsumstellung oder ein Sportprogramm, können zur Vorbeugung von Typ-2-Diabetes beitragen (3). Ein Vorteil dieser Interventionen gegenüber Arzneimitteln ist ihre große Sicherheit: es gibt nur wenige unerwünschte Nebenwirkungen. Darüber hinaus senken Lebensstilveränderungen nicht nur das Diabetesrisiko, sondern wirken sich beispielsweise auch günstig auf das Körpergewicht, den Blutdruck und die Lebensqualität aus (4; 5).

Auch Frauen, die einen Gestationsdiabetes hatten, können von einer Lebensstilintervention profitieren, sind aber mit konventionellen Programmen schwer zu erreichen. Zum Beispiel sind regelmäßige Sportgruppen oder Beratungstermine für junge Mütter ungeeignet. Dies führt dazu, dass klassische Interventionsprogramme häufig abgebrochen werden und erfolglos bleiben (6).

Wir haben deshalb die Smartphone-App „Triangle“ entwickelt, die eine auf Frauen nach Gestationsdiabetes zugeschnittene Lebensstilintervention ermöglichen soll. Die Bereiche Ernährung, Gewichtsmanagement, Sport und innere Balance (Psychologie) werden darin adressiert. Die App kombiniert Erinnerungs- und Motivationsfunktionen, Anleitung und Wissensvermittlung mit einem persönlichen Online-Coaching. Die Interventionsinhalte sind in Bausteine gegliedert und werden, beispielsweise abhängig vom Körpergewicht, an die einzelnen Nutzerinnen angepasst. Das Programm basiert auf verhaltenstheoretischen Modellen (7; 8), die Compliance und Motivation verbessern und nachhaltige Verhaltensänderungen gewährleistet sollen.

Standard-of-Care für Frauen nach Gestationsdiabetes ist in Deutschland ein oraler Glukosetoleranztest im ersten Jahr nach Entbindung. Bei einem auffälligen Ergebnis erfolgt dann häufig eine einmalige, nicht standardisierte Lebensstilberatung. Für das weitere Follow-Up gibt es derzeit keine eindeutigen Vorgaben (9).

# Studiendesign

Multizentrische, 2-armige, randomisierte, unverblindete Interventionsstudie (Pilotstudie)

*Interventionsarm*

6 Monate Lebensstilintervention in den Bereichen Ernährung, Gewichtsmanagement, Sport, innere Balance, vermittelt durch die Smartphone-App „Triangle“; App-Support und Online-Coaching für alle Studienteilnehmerinnen durch das Hauptstudienzentrum

*Kontrollarm*

Einmalige Lebensstilinformation zu Ernährung, Gewichtsmanagement, Bewegung, Innere Balance in Form eines Faltblatts; Die App-Intervention wird Teilnehmerinnen im Kontrollarm nach Abschluss der Studie in vollem Umfang kostenfrei angeboten.

# Studienziele

Es soll getestet werden, ob die Triangle-Intervention dazu beiträgt, dass die 5 klassischen Lebensstilziele der großen Diabetespräventionsstudien (DPP und DPS) erreicht werden. Für diese gibt es sehr gute Evidenz (3). Die 5 Ziele sind: ≥30min sportliche Aktivität moderater oder hoher Intensität an mindestens 5 Tagen pro Woche, Gewichtsreduktion ≥5%, ≤30% Energieaufnahme aus Fett, ≤10% Energieaufnahme aus gesättigten Fettsäuren, ≥15g Ballaststoffe pro 1000 kcal (6).

Mögliche weitere Interventionserfolge sollen explorativ, im Vergleich zur Kontrollgruppe, ausgewertet werden. Betrachtet werden insbesondere: Glukosetoleranz, Insulinempfindlichkeit, BMI, Fitness, Ernährungs- und Bewegungsverhalten und psychisches Wohlbefinden.

Durch diese Studie sollen auch geeignete Endpunkte für eine nachfolgende, konfirmatorische Interventionsstudie zum Wirksamkeitsnachweis der App identifiziert werde. Zusätzlich ermöglichen die Ergebnisse dieser Studie eine realistische Schätzung der Parameter für die Fallzahlschätzung (z.B. Standardabweichung) der konfirmatorischen Folgestudie.

Zusätzlich sollen Daten zu App-Akzeptanz und Nutzung gesammelt werden.

# Geplante Probandenzahlen

64 Teilnehmerinnen, davon circa 32 am Klinikum der Universität München und weitere 32 an den anderen aufgeführten Einrichtungen des Deutschen Zentrums für Diabetesforschung.

# Randomisierung

Randomisierung im Verhältnis 1:1, stratifiziert nach Zentrum.

# Rekrutierung

Frauen mit Gestationsdiabetes in einer vorausgegangenen Schwangerschaft werden vorwiegend aus dem Patientenstamm der beteiligten Kliniken, ggf. auch über niedergelassene Frauenärzte und Diabetologen, rekrutiert. Ein Infoflyer mit Studieninformationen wird ausgegeben. Ggf. können auch Anzeigen (gedruckt oder online) zur Rekrutierung verwendet werden.

Rekrutierungszeitraum: Juli bis Dezember 2017, ggf. Erweiterung bis einschl. März 2018

# Probandenvergütung

Fahrtkosten und ein Fitnessarmband (z. B. FitBit), zunächst leihweise, bei Compliance und erfolgreichem Studienabschluss dann dauerhaft (in Kontrollgruppe Fitnessarmband erst bei V2)

# Einschlusskriterien

- Ärztlich validierte Diagnose eines Gestationsdiabetes während einer Schwangerschaft, die mindestens 3, höchstens 18 Monate zurückliegt
- Rückbildungsphase nach Entbindung abgeschlossen, Sport aus geburtshilflicher Sicht uneingeschränkt möglich
- iPhone Nutzerin (iPhone 5 und neuere Versionen)
- gute Deutschkenntnisse

# Ausschlusskriterien

- Alter unter 18 Jahren
- Anamnestisch: Bestehende Schwangerschaft
- Schwangerschaft im nächsten halben Jahr geplant
- Kardiopulmonale Erkrankung, bei der ein Sportprogramm kontraindiziert ist
- Erkrankung des Bewegungsapparats, bei der ein Sportprogramm kontraindiziert ist
- Gastrointestinale Erkrankung, bei der eine Ernährungsumstellung kontraindiziert ist
- Psychiatrische Erkrankung, die einer psychotherapeutischen oder medikamentösen Therapie bedarf
- Andere schwerwiegende Erkrankung, bei der eine Lebensstilintervention kontraindiziert erscheint (nach Einschätzung des Studienarztes und in Absprache mit dem Hauptstudienzentrum)
- Stationäre Krankenhausbehandlung im nächsten halben Jahr geplant
- Alkohol- oder Drogenabusus
- Geplante Durchführung einer anderen Lebensstilintervention während des Studienzeitraums, einschließlich selbstdurchgeführter Programme
- Einnahme blutzuckersenkender Medikamente
- Diabetes mellitus (d.h. HbA1c ≥ 6,5%, Nüchtern-Plasmaglukose von ≥ 126 mg/dl oder oGTT-2-h-Wert im venösen Plasma

≥ 200 mg/dl)

# Abbruchkriterien

- Schwangerschaft
- Schwerwiegende Erkrankung, bei der eine Fortführung der Lebensstilintervention kontraindiziert erscheint (nach Einschätzung des Studienarztes und in Absprache mit dem Hauptstudienzentrum)
- Entzug des Einverständnisses der Studienteilnehmerin

# Interventionszeitraum

6 Monate

# Visitenübersicht

| **Visiten** | **Baselinevisite**  **V1** | **Interventions-Zeitraum** | **Abschlussvisite**  **V2** |
| --- | --- | --- | --- |
|  | 3^*)^-18 Monate nach Entbindung  ^*)^nach abgeschl. Rückbildung | 6 Monate | 6-8 Monate nach Interventions-  beginn |
| Überprüfung Ein-/Ausschlusskriterien | ● |  |  |
| Aufklärung/Einverständniserklärung | ● |  |  |
| Anamnese/Fragebögen 1 | ● |  |  |
| Anamnese/Fragebögen 2 |  |  | ● |
| Körperliche Untersuchung | ● |  | ● |
| Nüchternblutabnahme | ● |  | ● |
| 5-Punkt-oGTT | ● |  | ● |
| Bioimpedanzmessung (fakultativ) | ● |  | ● |
| Spiroergometrie (fakultativ) | ● |  | ● |
| Selbst auszufüllende Fragebögen |  | ● |  |

# Visitenbeschreibung

## Baselinevisite V1

Überprüfung von Ein- und Ausschlusskriterien

Aufklärung und schriftliche Einwilligung der Studienteilnehmerin

Nutzerregistrierung Triangle

*Anamnese / Fragebögen 1*

- Sozialanamnese (Bildung, Beruf usw.)
- Medizinische Vorgeschichte einschließlich Schwangerschaftsanamnese
- Aktuelle Medikation
- Familienanamnese
- Ernährung, insbesondere Anteil Fette, gesättigte Fette, Ballaststoffe
- Rauchen bzw. Exrauchen
- Validierte Fragebögen zu Lebensqualität, körperlicher Aktivität, Depression, Stress, Schlaf, Essverhalten (IPAQ, PSS-10, WHO-Five Well-being Index, EQ5-D Analogskala zum aktuellen Gesundheitszustand)
- Stillanamnese
- Nutzungsverhalten Smartphone, Apps, etc.

*Körperliche Untersuchung*

- Allg. körperliche Untersuchung (Auskultation Herz, Lunge, Ausschluss Beinödeme)
- Größe, Gewicht, Taillenumfang
- Ruheblutdruck, Puls

*Oraler Glukosetoleranztest (oGTT)*

75g Glucose oral; Blutentnahmen bei 0 (=Nüchternblutentnahme), 30, 60, 90, 120 Minuten aus liegendem peripherem Venenkatheter; Bestimmung von Plasmaglukose, Insulin

*Bei Nüchternblutentnahme zusätzlich:*

kleines Blutbild, Kreatinin, GPT, Gamma-GT, CRP, TSH, Triglyzeride, Cholesterin, LDL, HDL, HbA1c

*Bioimpedanzmessung (fakultativ; Bestimmung des Körperfettanteils)*

*Spiroergometrie (fakultativ)*

Stufenbelastung, Bestimmung von VO2peak (ml/min) und aeroben Schwellenwerten

## Abschlussvisite V2

*Anamnese*

- Aktuelles Allgemeinbefinden, spezifische Beschwerden
- Medikationsänderungen seit V1
- Erkrankungen seit V1
- Ernährung, insbesondere Anteil Fette, gesättigte Fette, Ballaststoffe
- Validierte Fragebögen zu Lebensqualität, körperlicher Aktivität (insbesondere mit Blick auf primären Endpunkt), Depression, Stress, Schlaf, Essverhalten (IPAQ, PSS-10, WHO-Five Well-being Index, EQ5-D Analogskala zum aktuellen Gesundheitszustand)
- Fragebögen zur subjektiven Einschätzung der Interventions-App (z. B. SUS, uMARS)

*Körperliche Untersuchung*

- Allg. körperliche Untersuchung (Auskultation Herz, Lunge, Ausschluss Beinödeme)
- Größe, Gewicht, Bauchumfang, Taillenumfang
- Ruheblutdruck, Puls

*Oraler Glukosetoleranztest (oGTT)*

75g Glucose oral; Blutentnahmen bei 0 (=Nüchternblutentnahme), 30, 60, 90, 120 Minuten aus liegendem peripherem Venenkatheter; Bestimmung von Plasmaglukose, Insulin

*Bei Nüchternblutentnahme zusätzlich:*

kleines Blutbild, Kreatinin, GPT, Gamma-GT, CRP, TSH, Triglyzeride, Cholesterin, LDL, HDL, HbA1c

*Bioimpedanzmessung (fakultativ; Bestimmung des Körperfettanteils)*

*Spiroergometrie (fakultativ)*

Stufenbelastung, Bestimmung von VO2peak (ml/min) und aeroben Schwellenwerten

# Laborbestimmungen

Alle Laborbestimmungen werden in den Zentrallabors der einzelnen Studienzentren durchgeführt.

# Erfassung von Nutzerdaten während der Intervention

- Subjektive Nutzerdaten durch validierte Fragebögen zu Akzeptanz, Zufriedenheit und Benutzerfreundlichkeit, wie uMARS, SUS
- Objektive Nutzerdaten durch Erfassung von Daten innerhalb der App, wie z.B. aktive Nutzungszeit, Häufigkeit und Dauer verwendeter App-Funktionen, Einstellungen innerhalb der App

# Auswertung der Studienergebnisse

Primärer Endpunkt: Welcher Anteil der Teilnehmerinnen erreicht 3 oder mehr der 5 klassischen DPP-Interventionsziele (6) nach 6 Monaten – Interventionsarm im Vergleich zum Kontrollarm?

| **Interventionsziel** | **1 Punkt, wenn** | |
| --- | --- | --- |
| Sportliche Aktivität mit moderater oder hoher Intensität | ≥ 150 Minuten pro Woche | |
| Ballaststoffe | ≥ 15g pro 1000 kcal | |
| Fettanteil | ≤ 30% Energie aus Fett | |
| Gesättigte Fettsäuren | ≤ 10% Energie aus Fett | |
| Körpergewicht | BMI v1 ≥ 23^*)^ | Gewicht v2 ≤ 95% von v1 |
|  | BMI v1 20 bis 22,9 | Gewicht v2 ≤ 100% von v1 |
|  | BMI v1 < 20 ^**)^ | Gewicht v2 ≤ 105% von v1 |
| ^*)^ In den aktuellen Leitlinien wird ein Grenzwert von 25 für die Gewichtsreduktion zur Diabetesprävention angegeben. Wir haben 23 gewählt, da die Zielgruppe deutlich jünger ist, als „übliche“ Präventionskohorten.  ^**)^ Im Rahmen der Intervention soll Muskelmasse aufgebaut werden. Deshalb wird bei sehr schlanken Frauen auch eine Gewichtszunahme während der Studie akzeptiert. | | |

Sekundäre Endpunkte:

- Änderung der AUC-Glucose des oGTT von V1 zu V2
- Änderung ISI V1 zu V2 (Insulin-Sensitivitätsindex nach Matsuda)
- Änderung DI V1 zu V2 (Disposition Index)
- Änderung BMI V1 zu V2 (stratifizierte Auswertung nach Ausgangs-BMI <≥ 23)
- Änderung VO2peak V1 zu V2
- Änderung Körperfettmasse V1 zu V2
- Änderung psychisches Wohlbefinden und Stressempfinden V1 zu V2

Die primäre Auswertung schließt alle randomisierten Teilnehmerinnen ein, die an V1 und V2 teilgenommen haben und für die die primären Endpunktdaten bei V2 erhoben werden konnten (Intention-To-Treat Analyse).

Zusätzlich werden zwei Per-Protokoll Analysen durchgeführt:

Per-Protokoll Gruppe 1: Teilnehmerinnen der Interventionsgruppe, die

- die Triangle-App installiert haben.
- mindestens eine Challenge aus mindestens zwei der drei Interventionsbereiche erfolgreich abgeschlossen haben.
- mindestens eine Chatnachricht geschrieben haben.
- mindestens einen Lexikonartikel geöffnet haben.
- mindestens einen Fragebogen innerhalb der App beantwortet haben.

Per-Protokoll Gruppe 2: Teilnehmerinnen der Per-Protokoll Gruppe 1, die in jedem der 6 Interventionsmonate

- mindestens eine Challenge aus mindestens zwei der drei Interventionsbereiche erfolgreich abgeschlossen haben.
- mindestens eine Chatnachricht geschrieben haben.
- mindestens einen Lexikonartikel geöffnet haben.
- mindestens einen Fragebogen innerhalb der App beantwortet haben (soweit vom Coach mindestens einer geschickt wurde).

Die sekundären Endpunkte werden zwischen Interventions- und Kontrollgruppe in explorativen Analysen verglichen, ggf. auch stratifiziert.

Außerdem werden folgende Daten ausgewertet:

- Nutzung der App anhand der innerhalb der App erhobenen Daten
- Subjektive Erfahrungen mit der App und wahrgenommener Einfluss auf das eigene Verhalten, z. B. Fragebogen „perceived impact on behavior“.

# Fallzahlschätzung

Der primäre Endpunkt für die Fallzahlberechnung ist ein binärer Endpunkt (erreichte Interventionsziele 0-2 versus 3-5). Entsprechend der vorliegenden Daten aus anderen Studien (6) wird in der Kontrollgruppe eine Erfolgsrate von 15% versus 50% in der Interventionsgruppe angenommen. Für die Auswertung wird ein unkorrigierter Chi-Quadrat Test mit einem Signifikanzniveau von 5% (2-seitig) und einer Power von 90% verwendet. Bei dem erwarteten Unterschied des primären Endpunkts müssen in beiden Gruppen jeweils 27 Probandinnen eingeschlossen werden. Bei einer angenommenen Abbruchrate von 15% müssen 32 Probandinnen pro Gruppe randomisiert werden.

# Zusätzliches Interventionsangebot außerhalb der randomisierten Studie für Teilnehmerinnen, bei denen im Rahmen der Baselinevisite V1 ein Diabetes mellitus diagnostiziert wird.

Wird bei V1 ein Diabetes mellitus neu diagnostiziert, kann die Teilnehmerin aufgrund der Ausschlusskriterien nicht an der randomisierten Studie teilnehmen. Um der betroffenen Frau dennoch Unterstützung anbieten zu können und um die Daten dieser prinzipiell relevanten Personengruppe nicht zu verlieren, erhalten alle diese Teilnehmerinnen unmittelbar das Angebot von Intervention und Abschlussvisite V2, analog zum oben geschilderten Interventionsarm. Diese Personengruppe geht jedoch nicht in Fallzahl und Auswertung der randomisierten Studie ein, sondern wird gesondert analysiert. Betroffene Teilnehmerinnen werden zudem angehalten, sich aufgrund der Diagnose Diabetes mellitus in ärztliche Überwachung und ggf. Behandlung zu begeben. Die App-Intervention dient in diesem Fall lediglich der zusätzlichen Unterstützung der Studienteilnehmerin.

# Mögliche Risiken der Studie / Versicherung

Die im Rahmen der Visiten durchgeführten Untersuchungen bergen vernachlässigbare Risiken für die Teilnehmerinnen. Die durchgeführte Intervention beinhaltet keine Empfehlungen, die über übliche Verhaltensweisen und freizeitsportliche Aktivitäten hinausgehen.

Für die Studie besteht eine Wegeunfallversicherung (Unfallversicherung für den Aufenthalt im Studienzentrum und den direkten Weg zwischen Wohnung und Studienzentrum; Unfall-Versicherung Nr. 50069894461, SV SparkassenVersicherung

Bahnhofstraße 69, 65185 Wiesbaden, Telefon: 0611 178-100, Telefax: 0611 178-109).

# Finanzierung des Projekts

Die Entwicklung der Triangle-App wurde durch die Else-Kröner-Fresenius-Stiftung gefördert. Die aktuelle Studie wird durch das Deutsche Zentrum für Diabetesforschung, eines der nationalen Gesundheitsforschungszentren, finanziert.

# Umgang mit Aufzeichnungen

## Datenschutz

Die erhobenen Daten werden vertraulich behandelt und pseudonymisiert (3-stellige Zahl) im jeweiligen Studienzentrum und im Hauptstudienzentrum archiviert. Neben konventionellen CRFs, die als Kopie per Post an das Hauptstudienzentrum geschickt werden, werden Online-Fragebögen über das System „Lime-Survey“ verwendet. Diese werden SSL-verschlüsselt an das Hauptstudienzentrum übertragen und enthalten zur Identifikation lediglich den Probandencode, keine persönlichen Daten. Zuordnungslisten der Probandencodes werden am jeweiligen lokalen Studienzentrum und am Hauptstudienzentrum aufbewahrt. Letzteres ist notwendig, um innerhalb der App erhobene Nutzungsdaten mit den übrigen Studiendaten zusammenführen zu können.

In der App werden keine persönlichen Daten der Teilnehmerinnen erfasst. Hier authentifiziert sich jede Nutzerin mit einem persönlichen App-Code, der vom Hauptstudienzentrum vergeben wird und vom Probandencode unterschiedlich ist. Zusätzlich zur Studienaufklärung erhält jede Teilnehmerin die Datenschutzerklärung der Triangle-App zur Unterschrift. Diese, sowie die App selbst, wird vom Datenschutzbeauftragten des Klinikums der Universität München vor Studienbeginn begutachtet und freigegeben.

## Archivierung der Aufzeichnungen in den Studienzentren

Die Einverständniserklärung, alle CRFs und Original-Dokumente müssen für 10 Jahre an den Studienzentren aufbewahrt werden.

## Handhabung und Weiterverarbeitung der Aufzeichnungen

Eine Veröffentlichung der Studienergebnisse erfolgt nur in aggregierter Form.

# Literaturverzeichnis

1. Kim C, Newton KM, Knopp RH. Gestational diabetes and the incidence of type 2 diabetes: a systematic review. Diabetes care. 2002; 25(10):1862-8.
2. Bellamy L, Casas JP, Hingorani AD, Williams D. Type 2 diabetes mellitus after gestational diabetes: a systematic review and meta-analysis. Lancet. 2009; 373(9677):1773-9.
3. Diabetes Prevention Program Research Group. Long-term effects of lifestyle intervention or metformin on diabetes development and microvascular complications over 15-year follow-up: the Diabetes Prevention Program Outcomes Study. Lancet Diabetes Endocrinol. 2015; 866-75
4. Nield L, Summerbell CD, Hooper L, Whittaker V, Moore H. Dietary advice for the prevention of type 2 diabetes mellitus in adults. Cochrane Database Syst Rev. 2008(3):CD005102.
5. Thomas D, Elliott EJ. Low glycaemic index, or low glycaemic load, diets for diabetes mellitus. Cochrane Database Syst Rev. 2009(1):CD006296.
6. O'Reilly SL, Dunbar JA, Versace V, Janus E, Best JD, Carter R, Oats JJ, Skinner T, Ackland M, Phillips PA, Ebeling PR, Reynolds J, Shih ST, Hagger V, Coates M, Wildey C; MAGDA Study Group. Mothers after Gestational Diabetes in Australia (MAGDA): A Randomised Controlled Trial of a Postnatal Diabetes Prevention Program.PLoS Med. 2016 Jul 26; 13(7):e1002092
7. Reed GR, Velicer WF, Prochaska JO, Rossi JS, Marcus BH. What makes a good staging algorithm: examples from regular exercise. Am J Heal Promot. 1997; 12(1):57-66.
8. Rosenstock IM, Strecher VJ, Becker MH. Social learning Theory and the Health Belief Model. Health Educ Q. 1988; 15(2):175-83.
9. Deutsche Diabetesgesellschaft: S3 Leitlinie Gestationsdiabetes; <http://www.deutsche-diabetes-gesellschaft.de/leitlinien/evidenzbasierte-leitlinien.html> (Abfrage 09.03.2017)

______________ ______________________

Datum PD Dr. med. A. Lechner

(Studienleiter)

Mit der Durchführung dieser Studie bin ich einverstanden.

______________ ______________________

Datum Prof. Dr. med. M. Reincke

(Direktor der Klinik)
