# Supplementary material for: A novel smartphone app to change risk behaviors of women after gestational diabetes: A randomized controlled trial
Source: PLoS One. 2022 Apr 27;17(4):e0267258. doi: 10.1371/journal.pone.0267258 (PMC9045614; doi:10.1371/journal.pone.0267258)
Supplement: S2 File — (DOCX) [file pone.0267258.s009.docx]

Study Protocol “Test Triangle“

Multicenter, randomized pilot study

to test an app-based lifestyle intervention

for women after gestational diabetes mellitus

v12.12.2017

# Main study center

Studienzentrum Diabetes – Diabetes Research Group

Medizinische Klinik und Poliklinik IV

Klinikum der Universität München

Ziemssenstr. 1

80336 München

Phone: +49 (0)89 – 4400-52234

Email: studienzentrum-diabetes@med.uni-muenchen.de

# Principal investigator

PD Dr. med. Andreas Lechner

Phone: +49 (0)89 – 4400-52185

Email: andreas.lechner@med.uni-muenchen.de

# Study coordinators

Anne Potzel (wissenschaftliche Mitarbeiterin)

Phone:  +49 (0)89 – 4400-53193

Email: [anne.potzel@med.uni-muenchen.de](mailto:anne.potzel@med.uni-muenchen.de)

Dr. med. Friederike Banning (Studienärztin)

Phone: +49 (0)89 – 4400-52159

Email: [friederike.banning@med.uni-muenchen.de](mailto:friederike.banning@med.uni-muenchen.de)

Louise Füeßl (Studienärztin)

Phone: +49 (0) 89 – 4400 52288

Email: louise.fueessl[@med.uni-muenchen.de](mailto:friederike.banning@med.uni-muenchen.de)

Vanessa Sacco (MFA)

Phone: +49 (0)89 – 4400-52234

Email: vanessa.sacco[@med.uni-muenchen.de](mailto:friederike.banning@med.uni-muenchen.de)

Janina Neubarth (wissenschaftliche Mitarbeiterin)

Phone:  +49 (0)89 – 4400-53193

Email: janina.neubarth@med.uni-muenchen.de

# Data management

Dipl.-Stat. Marietta Rottenkolber

Phone: +49 (0)89 – 4400-53193

Email: marietta.rottenkolber@med.uni-muenchen.de

# Additional study centers

Institut für Diabetesforschung und Metabolische Erkrankungen, Universitätsklinikum Tübingen, Klinik für Innere Medizin IV, Otfried-Müller-Straße 10, 72076 Tübingen

Universitätsklinikum Dresden, Medizinischen Klinik und Poliklinik III, Fetscherstr. 74, 01307 Dresden

Deutsches Diabeteszentrum (DDZ), Institut für Klinische Diabetologie, Leibniz Zentrum für Diabetesforschung, Heinrich-Heine-Universität Düsseldorf, Auf´m Hennekamp 65, 40225 Düsseldorf

# Clinical background

Gestational diabetes mellitus (GDM) is a temporary disturbance of glucose metabolism occurring in late pregnancy. With standard-of-care treatment, the prognosis of this disease for mother and child is excellent.

However, women who develop GDM carry a substantially increased risk to develop type 2 diabetes later in life. Type 2 diabetes is a chronic and often difficult to treat metabolic disease with life threatening complications. The prevalence of type 2 diabetes 10 years after GDM is between 20 and 70% (1; 2).

Lifestyle interventions, such as weight loss, optimized nutrition and exercise, can contribute to the prevention of type 2 diabetes (3). An advantage of these interventions over drug therapy is their excellent safety profile. Unwanted side effects are rare. In addition, lifestyle interventions do not only lower the risk of type 2 diabetes. They also have beneficial effects on body weight, blood pressure and quality of life (4; 5).

Woman after GDM can also benefit from lifestyle change but are difficult to reach by conventional intervention programs. Fixed schedules for counseling and exercise, for example, are difficult to fulfill for young mothers. Therefore, conventional intervention programs are often terminated early by the women and remain unsuccessful (6).

We therefore developed a smartphone app „Triangle”, which facilitates a lifestyle intervention program specifically targeted to women after GDM. It addresses the areas nutrition, weight management, exercise and inner balance. The app combines reminders, motivation and education with one-on-one online coaching. To permit personalization, the intervention is split into packages, which can be selected specifically for individual women, e.g. based on body weight. The intervention program is based on theories of behavior change (7; 8) to facilitate lasting change.

Standard of care for women after GDM in Germany is an oral glucose tolerance test during the first year postpartum. If this test shows elevated values, non-standardized, one-time lifestyle counseling is often offered. However, no guidelines exist to standardize further follow-up (9).

# Study design

Multicenter, 2-arm, randomized, unblinded intervention study

*Intervention*

6 months of lifestyle intervention in the areas of nutrition, weight management, exercise and inner balance, delivered through the smartphone app “Triangle”; app support and online coaching by the main study center for all participants in this arm.

*Control*

One-time lifestyle information provided in the form of a leaflet. The app-intervention is offered to the participants in the control arm after completion of the study.

# Study aims

The study tests whether the Triangle intervention fosters reaching the 5 classic lifestyle goals of the Diabetes Prevention Program (DPP) (3). These 5 goals are: ≥30 minutes of moderate to high intensity exercise on at least 5 days per week, weight reduction ≥5%, ≤30% of energy from fat, ≤10% of energy from saturated fat, ≥15g of fiber per 1000 kcal of energy (6).

Additional intervention targets are also explored in comparison to the control arm. These are glucose tolerance, insulin sensitivity, BMI, physical fitness, overall nutrition and physical activity, as well as psychologic wellbeing.

This study is also meant to provide information on suitable endpoints and required sample size for a future confirmatory trial. Finally, data on app acceptance and usage are collected.

# Intended number of study participants

64 participating women (32 recruited at the main study center and 32 at the other study centers)

# Randomization

1:1 randomization, stratified by study center

# Recruitment of study participants

Women after GDM are preferentially recruited from the patient base of the participating centers, potentially also through obstetricians and diabetologists in private practice. A study information leaflet is provided. Additionally, advertisements (in print or online) may be placed to support recruitment.

Intended recruitment period: July - December 2017, potentially with extension until March 2018

# Reimbursement of study participants

Cost of travel to the study visits is reimbursed and a fitness tracker is provided free of charge (at V1 to the intervention group, after V2 to the control group)

# Inclusion criteria

- Physician-validated diagnosis of GDM during a preceding pregnancy with a life birth, delivery 3-18 months prior to study inclusion
- Postpartum regeneration completed
- iPhone user (version 5 or newer)
- fluent in German

# Exclusion criteria

- <18 years of age
- Current pregnancy
- Pregnancy planned during the following 6 months
- Cardiopulmonary disease precluding an exercise program
- Musculo-skeletal disease precluding an exercise program
- Gastrointestinal disease precluding dietary intervention
- Psychiatric disease requiring psychotherapy or medical treatment
- Other severe disease precluding lifestyle intervention (judgement of study physician in concurrence with main study center)
- Planed inpatient hospital stay during the following 6 months
- Alcohol or drug abuse
- Planned alternative lifestyle intervention during the study period
- Glucose lowering medication
- Diabetes mellitus (HbA1c ≥ 6,5%, fasting plasma glucose ≥ 126 mg/dl or oGTT 2h plasma glucose

≥ 200 mg/dl)

# Reasons for termination of study participation

- Pregnancy
- Severe disease precluding further lifestyle intervention (judgement of study physician in concurrence with main study center)
- Withdrawal of consent

# Intervention period

6 months

# Summary of study visits

| **Visits** | **Baseline**  **V1** | **Intervention Period** | **Final**  **V2** |
| --- | --- | --- | --- |
|  | 3^*)^-18 months postpartum  ^*)^after completed postpartal regeneration | 6 months | 6-8 months after start of intervention |
| Check inclusion/exclusion criteria | ● |  |  |
| Obtain informed consent | ● |  |  |
| History/questionnaires 1 | ● |  |  |
| History/questionnaires 2 |  |  | ● |
| Physical examination | ● |  | ● |
| Fasting blood draw | ● |  | ● |
| 5 point oGTT | ● |  | ● |
| Bioimpedance measurements (not mandatory) | ● |  | ● |
| Ergospirometry (not mandatory) | ● |  | ● |
| Self-administered questionnaires |  | ● |  |

# Description of study visits

## Baseline V1

Check of Inclusion/Exclusion criteria

Informed consent

Randomization

Registration with the Triangle app in the intervention arm

*History / Questionnaires 1*

- Social status (education, occupation, …)
- Medical history
- Current medication
- Family history
- Nutritional pattern
- Smoking
- Validated questionnaires regarding quality of life, physical activity, depression, stress, sleep, eating habits (IPAQ, PSS-10, WHO-Five Well-being Index, EQ5-D visual scale)
- Breast feeding history
- Pattern of smartphone and app use

*Physical examination*

- General physical examination of circulation, heart and lungs
- Height, weight, waist circumference
- Resting blood pressure and pulse

*Oral glucose tolerance test (oGTT)*

75g oral glucose oral; blood draws at 0 (fasting), 30, 60, 90, 120 minutes from indwelling peripheral venous catheter, measurement of plasma gluose and serum insulin

*Additional measurements from fasting blood draw:*

Complete blood count, creatinine, GPT, Gamma-GT, CRP, TSH, triglycerides, cholesterol, LDL, HDL, HbA1c

*Bioimpedance measurement (not mandatory; to determine body fat content)*

*Ergospirometry (not mandatory)*

Stepwise protocol, determination of VO2peak (ml/min) und aerobic thresholds

## Abschlussvisite V2

*History / questionnaires 2*

- Current wellbeing, specific acute complaints
- Changes in medication since V1
- New diagnoses since V1
- Nutritional pattern
- Smoking
- Validated questionnaires regarding quality of life, physical activity, depression, stress, sleep, eating habits (IPAQ, PSS-10, WHO-Five Well-being Index, EQ5-D visual scale)
- Questionnaires regarding the app in the intervention arm (z. B. SUS, uMARS)

*Physical examination*

- General physical examination of circulation, heart and lungs
- Height, weight, waist circumference
- Resting blood pressure and pulse

*Oral glucose tolerance test (oGTT)*

75g oral glucose oral; blood draws at 0 (fasting), 30, 60, 90, 120 minutes from indwelling peripheral venous catheter, measurement of plasma glucose and serum insulin

*Additional measurements from fasting blood draw:*

Complete blood count, creatinine, GPT, Gamma-GT, CRP, TSH, triglycerides, cholesterol, LDL, HDL, HbA1c

*Bioimpedance measurement (not mandatory; to determine body fat content)*

*Ergospirometry (not mandatory)*

Stepwise protocol, determination of VO_2_peak (ml/min) und aerobic thresholds

# Laboratory chemistry

All assays are run in the central laboratory of the respective study center.

# Collection of user data during the intervention

- Subjective user data through in-app questionnaires
- Objective user data (active use time of app, use of specific features within the app, app settings

# Analysis of study results

Primary endpoint: Proportion of study participants reaching 3 or more of the 5 classic DPP lifestyle goals after 6 months – comparison of intervention and control arm.

| **Lifestyle goals** | **Scores 1 point if** | |
| --- | --- | --- |
| Exercise of moderate or high intensity | ≥ 150 minutes per week | |
| Fiber | ≥ 15g per 1,000 kcal | |
| Fat | ≤ 30% of energy intake | |
| Saturated fatty acids | ≤ 10% of energy intake | |
| Body weight | BMI v1 ≥ 23^*)^ | weight v2 ≤ 95% of v1 |
|  | BMI v1 20 - 22,9 | weight v2 ≤ 100% of v1 |
|  | BMI v1 < 20 ^**)^ | weightt v2 ≤ 105% of v1 |
| ^*)^ Generally, a recommendation for weight loss for diabetes prevention is given at a BMI of 25 or higher. Here however, we chose a cutoff of 23 because of the young age of the target group.  ^**)^ Building up muscle mass is intended. Therefore, some weight gain is accepted in very lean women. | | |

Secondary endpoint:

- Change of AUC glucose in the oGTT V1 to V2
- Change of ISI V1 to V2 (insulin sensitivity index according to Matsuda/deFronzo)
- Change of DI V1 to V2 (Disposition Index)
- Change of BMI V1 to V2 (stratified according to baseline BMI < ≥ 23)
- Change of VO2peak V1 to V2
- Change of body fat mass V1 to V2
- Change of psychologic wellbeing and perceived stress V1 to V2

The primary analysis includes all randomized women who participated in V1 and V2 and for whom the primary endpoint could be determined at V2 (modified intention-to-treat analysis).

Additionally, two per-protocol groups of the intervention arm may be analyzed:

Per-protocol group 1: All women who, during the whole study period,

- installed the Triangle app.
- completed at least one challenge.
- wrote at least one message to the coach.
- read at least one article in the app dictionary.
- answered at least one in-app questionnaire.

Per-protocol group 2: All women who, in each month of the intervention period,

- completed at least one challenge.
- wrote at least one message to the coach.
- read at least one article in the app dictionary.
- answered at least one in-app questionnaire.

The secondary endpoints are compared between the intervention and the control arm in an exploratory manner, partly in stratified analyses.

Additional data collected:

- App use from data collected via the app
- Subjective experiences with the app and perceived impact on own behavior

# Sample size calculation

The primary, binary endpoint for the sample size calculation is reaching 3-5 (success) vs. 0-2 (no success) of the DPP lifestyle goals. Based on previous studies (6), we estimate a 15% success rate in the control arm vs. a 50% success rate in the intervention arm. Using an uncorrected Chi^2^ test with a 2-sided level of significance of 5% and a power of 90%, 27 participants per arm need to be analyzed. Assuming a loss-of-follow-up of 15 %, 32 participants per arm need to be randomized.

# Intervention with a diagnosis of diabetes at V1

If diabetes mellitus is diagnosed at V1, trial participation is not permitted. Nevertheless, such women are offered to participate in the Triangle intervention outside of the trial. Additionally, they are advised to seek specialized diabetologic care.

# Potential risks of participation / insurance

The trial visits carry very small risks for the participants and the intervention does not include recommendations that go beyond regular behavior and leisure exercise.

Insurance for injuries occurring during or on the way to the study visits is provided.

# Project finances

The development of the Triangle app has been funded by the Else-Kröner-Fresenius-Stiftung. The trial is funded by the German Center for Diabetes Research.

# Handling of study data

## Data protection

Data is linked to study participants via a random code and stored on paper as well as digitally. Data transfer uses SSL encryption. Data collected at the study visits and through the app are brought together at the main study center.

The app does not collect personal data or any data on the device outside of the app itself. Each participant in the intervention arm consents individually to the data handling policy of the app, which has also been approved by the data protection officer of the LMU hospital in Munich, Germany.

## Storage of study data

All data and study related information are stored for 10 years at the main study center.

## Use of study data

Publication of study data only occurs in aggregated form, not on an individual basis.

# References

1. Kim C, Newton KM, Knopp RH. Gestational diabetes and the incidence of type 2 diabetes: a systematic review. Diabetes care. 2002; 25(10):1862-8.
2. Bellamy L, Casas JP, Hingorani AD, Williams D. Type 2 diabetes mellitus after gestational diabetes: a systematic review and meta-analysis. Lancet. 2009; 373(9677):1773-9.
3. Diabetes Prevention Program Research Group. Long-term effects of lifestyle intervention or metformin on diabetes development and microvascular complications over 15-year follow-up: the Diabetes Prevention Program Outcomes Study. Lancet Diabetes Endocrinol. 2015; 866-75
4. Nield L, Summerbell CD, Hooper L, Whittaker V, Moore H. Dietary advice for the prevention of type 2 diabetes mellitus in adults. Cochrane Database Syst Rev. 2008(3):CD005102.
5. Thomas D, Elliott EJ. Low glycaemic index, or low glycaemic load, diets for diabetes mellitus. Cochrane Database Syst Rev. 2009(1):CD006296.
6. O'Reilly SL, Dunbar JA, Versace V, Janus E, Best JD, Carter R, Oats JJ, Skinner T, Ackland M, Phillips PA, Ebeling PR, Reynolds J, Shih ST, Hagger V, Coates M, Wildey C; MAGDA Study Group. Mothers after Gestational Diabetes in Australia (MAGDA): A Randomised Controlled Trial of a Postnatal Diabetes Prevention Program.PLoS Med. 2016 Jul 26; 13(7):e1002092
7. Reed GR, Velicer WF, Prochaska JO, Rossi JS, Marcus BH. What makes a good staging algorithm: examples from regular exercise. Am J Heal Promot. 1997; 12(1):57-66.
8. Rosenstock IM, Strecher VJ, Becker MH. Social learning Theory and the Health Belief Model. Health Educ Q. 1988; 15(2):175-83.
9. Deutsche Diabetesgesellschaft: S3 Leitlinie Gestationsdiabetes; <http://www.deutsche-diabetes-gesellschaft.de/leitlinien/evidenzbasierte-leitlinien.html> (Abfrage 09.03.2017)

______________ ______________________

date PD Dr. med. A. Lechner

(PI)

Consenting to the conduction of this trial

______________ ______________________

date Prof. Dr. med. M. Reincke

(director of department)
